# Intercellular hif1α reprograms mammary progenitors and myeloid immune evasion to drive high-risk breast lesions

**DOI:** 10.1172/JCI164348

**Published:** 2023-04-17

**Authors:** Irene Bertolini, Michela Perego, Yulia Nefedova, Cindy Lin, Andrew Milcarek, Peter Vogel, Jagadish C. Ghosh, Andrew V. Kossenkov, Dario C. Altieri

**Affiliations:** 1 Immunology, Microenvironment and Metastasis Program, The Wistar Institute, Philadelphia, Pennsylvania, USA.; 2 Department of Pathology, St. Jude Children’s Research Hospital, Memphis, Tennessee, USA.; 3 Gene Expression and Regulation Program and; 4 Center for Systems and Computational Biology, The Wistar Institute, Philadelphia, Pennsylvania, USA.

**Keywords:** Cell Biology, Oncology, Breast cancer, Hypoxia, Neutrophils

## Abstract

The origin of breast cancer, whether primary or recurrent, is unknown. Here, we show that invasive breast cancer cells exposed to hypoxia release small extracellular vesicles (sEVs) that disrupt the differentiation of normal mammary epithelia, expand stem and luminal progenitor cells, and induce atypical ductal hyperplasia and intraepithelial neoplasia. This was accompanied by systemic immunosuppression with increased myeloid cell release of the alarmin S100A9 and oncogenic traits of epithelial-mesenchymal transition, angiogenesis, and local and disseminated luminal cell invasion in vivo. In the presence of a mammary gland driver oncogene (MMTV-PyMT), hypoxic sEVs accelerated bilateral breast cancer onset and progression. Mechanistically, genetic or pharmacologic targeting of hypoxia-inducible factor-1α (HIF1α) packaged in hypoxic sEVs or homozygous deletion of S100A9 normalized mammary gland differentiation, restored T cell function, and prevented atypical hyperplasia. The transcriptome of sEV-induced mammary gland lesions resembled luminal breast cancer, and detection of HIF1α in plasma circulating sEVs from luminal breast cancer patients correlated with disease recurrence. Therefore, sEV-HIF1α signaling drives both local and systemic mechanisms of mammary gland transformation at high risk for evolution to multifocal breast cancer. This pathway may provide a readily accessible biomarker of luminal breast cancer progression.

## Graphical abstract

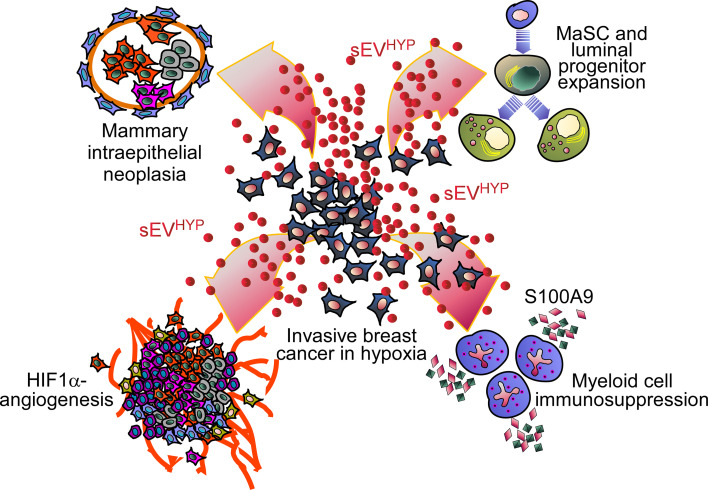

## Introduction

Breast cancer is the second most common malignancy diagnosed in women, resulting in over 43,000 deaths a year in the United States alone (1). Despite progress in early diagnosis and therapy (2), local and distant disease recurrences, often involving colonization of distant organs, i.e., metastasis (3), continue to account for treatment failure and abbreviated patient survival (4). A critical step in this process is the disruption of a complex hierarchy of mammary gland differentiation (5), which introduces oncogenic traits in multiple cellular subsets (6), including stem and progenitor cell compartments (7). However, deregulated mammary gland differentiation is insufficient, alone, to drive disease recurrence, and reprogramming of a host of seemingly normal cell types in the microenvironment (8) is now viewed as essential to expand tumor heterogeneity, enable treatment resistance, and confer metastatic competence (9).

Key mechanisms in the tumor-microenvironment crosstalk have been uncovered (8), and epithelial-mesenchymal transition (EMT) (10), metabolic reprogramming (11), and resistance to cell death (12) are invariably associated with late-stage, metastatic breast cancer (3). This often involves potent oncogenic functions of hypoxia-inducible factor-1α (HIF1α) (13), which orchestrates an adaptive response to acute, chronic, and cyclic hypoxia conditions in a breast cancer microenvironment (14) to confer treatment resistance and poor patient outcome (15). In this context, hypoxia-associated transcriptional programs have been linked to breast cancer immunosuppression (16). This may involve a collection of mechanisms (17), mostly driven by myeloid cell types, including myeloid-derived suppressor cells (18), to prevent intratumoral infiltration of effector T cells (19), reprogram cytokine responses (20), and exploit stress response effectors such as the S100 family of “alarmins” (21) for immune evasion. However, a role of these pathways in earlier stages of epithelial cell transformation is unclear, and whether they contribute to breast premalignant lesions (22) and “field cancerization” (23), two high-risk conditions that predispose to multifocal and recurrent disease, has not been investigated.

Released by virtually all cell types, small (40–150 nm) extracellular vesicles (sEVs) (24) have emerged as important mediators of intercellular communication in a breast cancer microenvironment (25), influencing the continuum of tumorigenesis, from oncogenic transformation (26) to metastatic competence (27). In addition to tumor cells, this pathway affects multiple components of the microenvironment, including the “normal” mammary epithelium, driving mitochondria-fueled cell movements (28) and NF-κB–dependent inflammation (29). Importantly, these responses are specific for a population of sEVs released by tumor cells under conditions of hypoxia, a major determinant of breast cancer progression and poor patient outcome (30).

In this study, we asked whether breast cancer–derived hypoxic sEVs alter mammary gland differentiation in vivo (7), potentially contributing to early stages of breast tumorigenesis and multifocal disease (22).

## Results

### Breast cancer–derived hypoxic sEVs induce mammary gland premalignant lesions.

We began this study by testing the role of sEVs released by breast cancer cells in normal mammary gland morphogenesis in vivo. For these experiments, we isolated sEVs from metastatic (AT3) or non-metastatic (EO771) mouse mammary adenocarcinoma cell lines under conditions of normoxia (sEV^NORM^, 20% O_2_) or hypoxia (sEV^HYP^, 1% O_2_). For all the experiments, sEVs isolated from non-conditioned medium were used as control (sEV^CTRL^). Consistent with Minimal Information for Studies of Extracellular Vesicles guidelines (31), sEV^NORM^ or sEV^HYP^ collected from AT3 or EO771 cells had comparable size distribution and yield (Supplemental Figure 1A; supplemental material available online with this article; https://doi.org/10.1172/JCI164348DS1), zeta potential (Supplemental Figure 1B), and expression of the sEV-associated tetraspanin proteins CD81, CD63, and CD9 (Supplemental Figure 1C). Similar results were obtained at the protein level, where sEVs isolated from AT3 or EO771 cell types contained the sEV-associated markers flotillin-1, TSG101, and CD9, but not the Golgi-related protein GM130 or the ER-related protein calnexin (Supplemental Figure 1D), by Western blotting.

**Figure 1:**
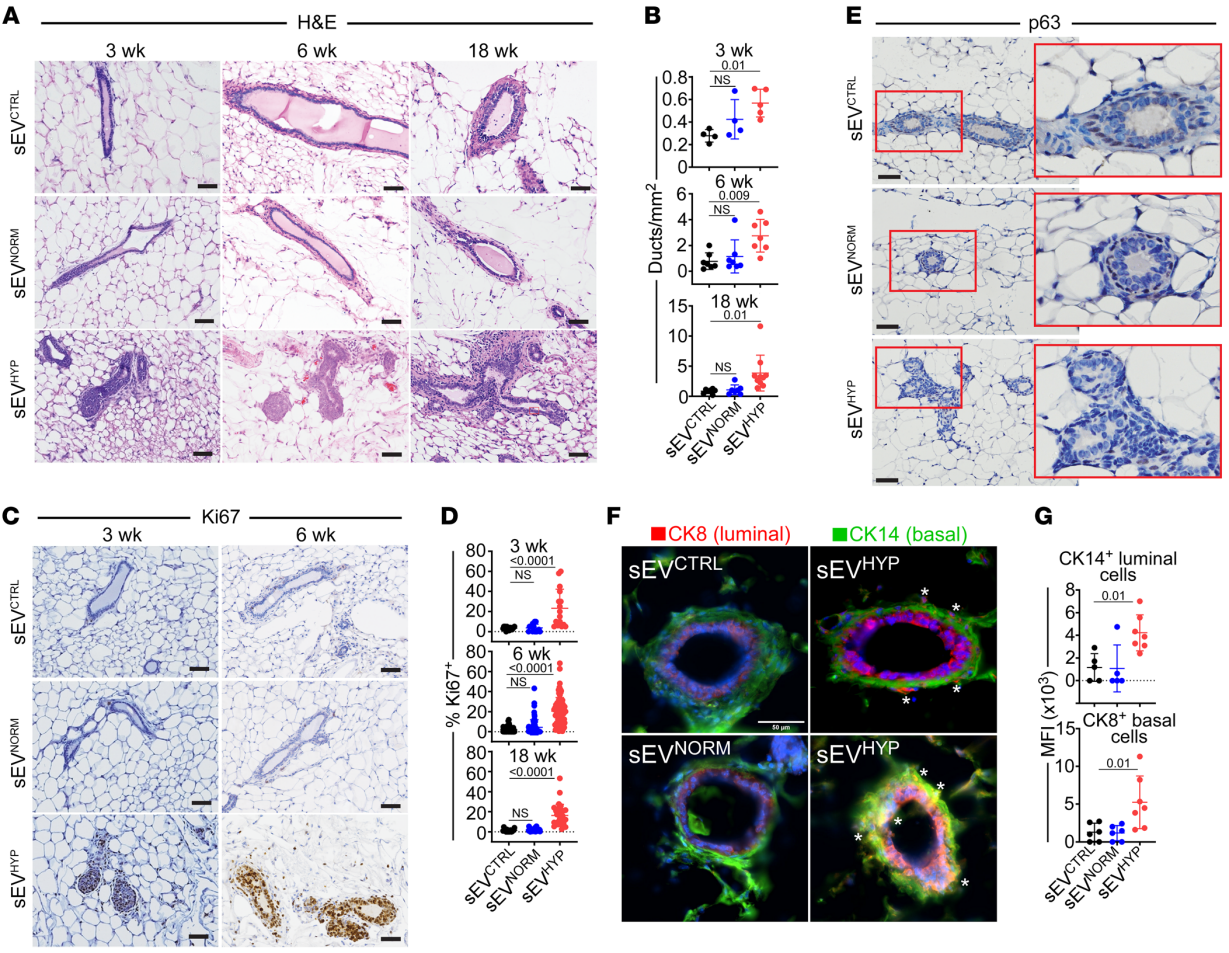
Hypoxic sEVs (sEV^HYP^) induce mammary gland hyperplasia in vivo. (**A** and **B**) AT3 cell–derived sEVs were injected in the abdominal mammary gland of immunocompetent C57BL/6 female mice, and tissue samples were analyzed after 3, 6, and 18 weeks by H&E staining and immunohistochemistry (IHC) (**A**, representative images) with quantification of the number of mammary gland ducts (**B**). Scale bars: 100 μm. Mean ± SD (*n* = 4). (**C** and **D**) Mammary gland tissues as in **A** were stained with an antibody against Ki67 by IHC (**C**, representative images), and the percentage of positive cells was quantified (**D**). Scale bars: 100 μm. Mean ± SD (*n* = 4). (**E**) Mammary gland tissues were analyzed for p63 reactivity after 6 weeks by IHC. Representative images (*n* = 4). Scale bars: 100 μm. Red boxes, magnification of indicated areas. (**F**) Mammary gland tissues were analyzed after 6 weeks for expression of luminal marker cytokeratin 8 (CK8, red) and basal marker cytokeratin 14 (CK14, green) by immunofluorescence microscopy. Asterisks, mislocalized apical-basal markers in sEV^HYP^-treated mammary gland. Representative images (*n* = 5). Scale bar: 50 μm. (**G**) Conditions were as in **F**, and mammary glands injected with the various sEVs were quantified for apical-basal mislocalization of CK8^+^ or CK14^+^ cells. MFI, mean fluorescence intensity. Mean ± SD. For all panels, numbers correspond to *P* values by 1-way ANOVA with Tukey’s multiple-comparison test.

Under these conditions, a single injection of AT3 cell–derived sEV^HYP^ was sufficient to induce a broad spectrum of histologic alterations in the mammary gland of female C57BL/6 immunocompetent mice (*n* = 6 mice per group) (Supplemental Table 1), detectable 3 weeks after sEV administration and persisting throughout 18 weeks of observation (Figure 1, A and B). Histologically, the lesions included multifocal moderate to marked hyperplasia of the mammary epithelium and low-grade, marked multifocal intraepithelial neoplasia, with increased number and branching of distorted mammary ducts lined by multiple, disordered epithelial layers with hyperchromatic nuclei (Figure 1, A and B). Consistent with hyperplastic morphology, sEV^HYP^ treatment increased mammary duct proliferation at all time points tested, in vivo, by Ki67 staining and immunohistochemistry (Figure 1, C and D). In contrast, injection of C57BL/6 female mice with AT3 cell–derived sEV^NORM^ or sEV^CTRL^ (Figure 1, A–D) or sEV^HYP^ isolated from non-metastatic EO771 cells (*n* = 3 mice per group) (Supplemental Figure 1, E and F) did not affect mammary gland morphogenesis, number of ducts, or Ki67-associated cell proliferation.

Next, we examined the effect of sEV^HYP^ on the expression and topography of mammary gland differentiation markers. In these experiments, injection of AT3 cell–derived sEV^HYP^ caused mislocalization (Figure 1E and Supplemental Figure 1G) and reduced expression (Supplemental Figure 1H) of the myoepithelial proteins p63 (Figure 1E) and SMMHC (Supplemental Figure 1G) in comparison with control mice. Mammary gland polarity was also disrupted under these conditions, with mislocalization of luminal (cytokeratin 8) and basal (cytokeratin 14) markers (Figure 1, F and G). In contrast, injection of mammary glands with AT3 cell–derived sEV^NORM^ or EO771 cell–derived sEV^HYP^ had no effect on the polarity and spatial distribution of basal versus luminal markers (Figure 1, F and G) or the expression and localization of myoepithelial proteins (Supplemental Figure 1I).

### Mammary epithelial metabolic reprogramming mediated by sEV^HYP^.

**Figure 2:**
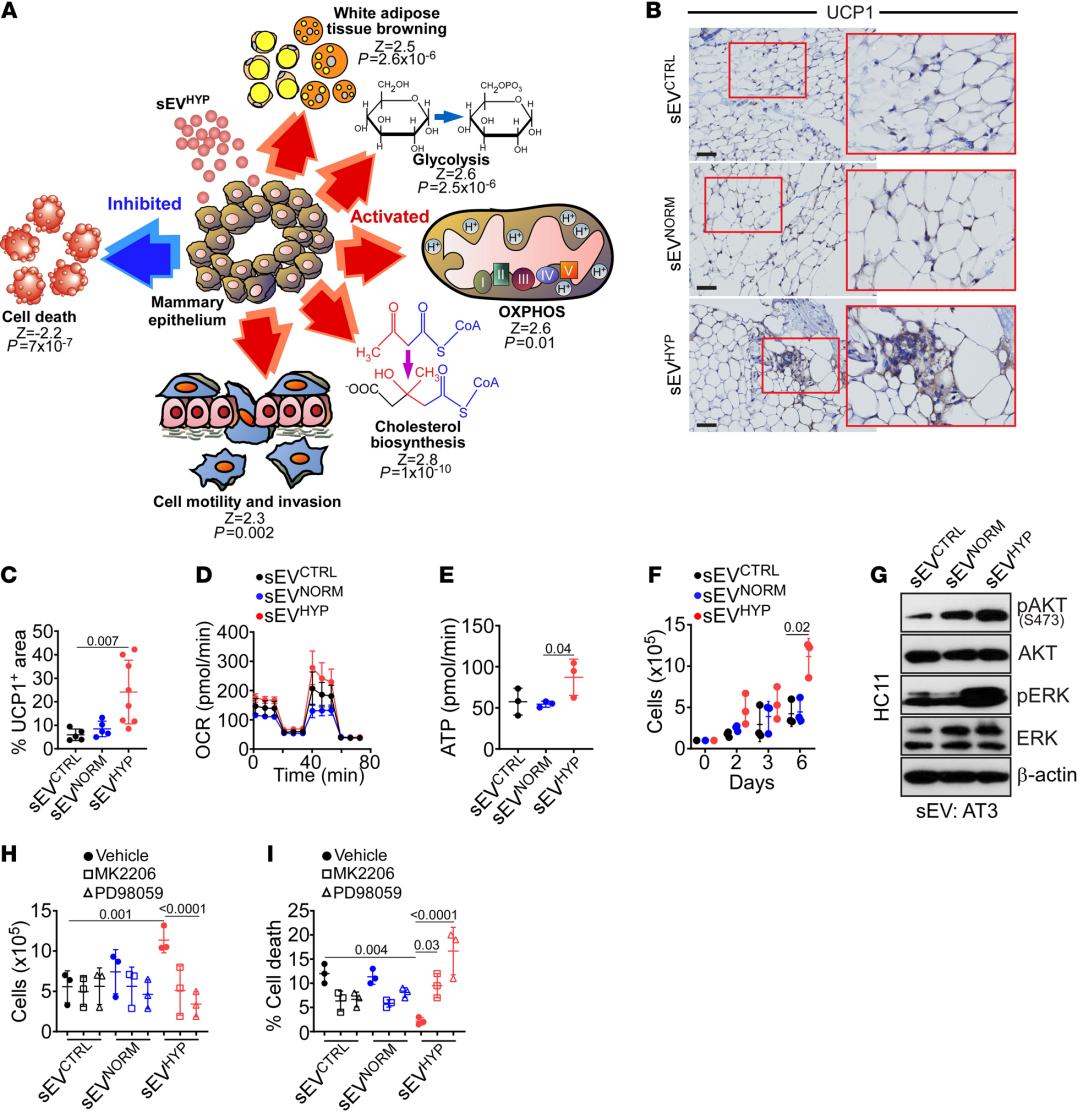
sEV^HYP^ regulation of mammary epithelium bioenergetics. (**A**) Schematic diagram of gene pathways upregulated (red) or downregulated (blue) in mammary glands of C57BL/6 female mice injected with sEV^HYP^ by RNA-Seq and Ingenuity Pathway Analysis. *Z* scores and *P* values for each modulated gene pathway are indicated. OXPHOS, oxidative phosphorylation. (**B** and **C**) Mammary glands harvested 6 weeks after sEV injection were analyzed for UCP-1 expression by IHC (**B**, representative images) and quantified (**C**). Red boxes, magnification of indicated areas. Scale bars: 100 μm. Mean ± SD (*n* = 5). (**D**) Primary mammary epithelial HC11 cells were incubated with AT3 cell–derived sEVs and analyzed for oxygen consumption rates (OCR) on an Agilent Seahorse flux analyzer. Mean ± SD (*n* = 3). (**E**) The conditions were as in **D**, and the rate of ATP production was quantified. Mean ± SD (*n* = 3). (**F**) HC11 cells were incubated with AT3 cell–derived sEVs and analyzed for cell proliferation by direct cell counting. Mean ± SD (*n* = 3). (**G**) HC11 cells as in **F** were analyzed after 3 days by Western blotting. p, phosphorylated. (*n* = 3.) (**H** and **I**) sEV-treated HC11 cells were incubated with vehicle (closed circles), Akt inhibitor MK2206 (1 μM, open squares), or ERK inhibitor PD98059 (10 μM, open triangles) and analyzed for cell proliferation (**H**) or cell death (**I**) after 7 days by direct cell counting. Mean ± SD (*n* = 3). For all panels, numbers correspond to *P* values by 1-way ANOVA with Tukey’s multiple-comparison test.

Based on these data, we next profiled the transcriptome of mammary glands exposed to sEVs for 6 weeks, by RNA sequencing (RNA-Seq) (*n* = 2–3 animals per group). Here, sEV^HYP^ affected the expression of 406 and 740 genes compared with sEV^CTRL^ or sEV^NORM^, respectively (*P* < 0.05). Of these, sEV^HYP^ modulated 154 unique genes with the top 30 upregulated (Supplemental Figure 2A) or downregulated (Supplemental Figure 2B) genes exhibiting fold change (FC) differences of 1.78–14.5 (*P* < 0.05). Bioinformatics analysis of this data set revealed that sEV^HYP^ treatment upregulated glycolytic and oxidative phosphorylation pathways, cholesterol biosynthesis, and white adipose tissue browning (Figure 2A). Regulators of cell motility and cell invasion were also increased, whereas cell death pathways of apoptosis and necroptosis were suppressed (Figure 2A).

In validation experiments, injection of mouse mammary glands with AT3 cell–derived sEV^HYP^, but not sEV^NORM^, increased the expression of uncoupling protein-1 (UCP-1), a mitochondrial effector of brown adipocyte bioenergetics, by immunohistochemistry (Figure 2, B and C). Conversely, sEV^HYP^ injected from non-metastatic EO771 cells had no effect on UCP-1 expression compared with controls (Supplemental Figure 2C) in vivo. To quantify the effect of sEV^HYP^ on metabolism, we next used the model of primary mouse mammary epithelial HC11 cells. sEV^HYP^ treatment increased HC11 oxygen consumption rates (Figure 2D), a marker of oxidative phosphorylation, resulting in heightened basal respiration (sEV^NORM^, 69.9 ± 3.9 pmol/min; sEV^HYP^, 113.4 ± 23.2 pmol/min; *P* = 0.02) and greater ATP production, in comparison with controls (Figure 2E). Consistent with increased glycolysis from RNA-Seq data, sEV^HYP^ stimulation also lowered glucose content, increased extracellular acidification rates (sEV^NORM^, 12.4 ± 1.4 milli pH/min, and sEV^HYP^, 18.4 ± 1.3 milli pH/min**)** at 72 minutes), and elevated lactate production (Supplemental Figure 2D).

In line with increased energy production, AT3 cell–derived sEV^HYP^, but not sEV^NORM^, stimulated HC11 cell proliferation in a time-dependent manner (Figure 2F), accompanied by phosphorylation, i.e., activation of Akt and ERK kinases, by Western blotting (Figure 2G). Mechanistically, small-molecule inhibition of Akt (MK2206) or ERK (PD98059) reversed the increase in cell proliferation mediated by sEV^HYP^ (Figure 2H) and induced HC11 cell death (Figure 2I) in comparison with controls. In contrast, AT3 cell–derived sEV^NORM^ (Figure 2, F and G) or EO771 cell–derived sEV^HYP^ or sEV^NORM^ (Supplemental Figure 2, E and F) did not affect HC11 cell proliferation, cell death, or ERK/Akt kinase activation.

### sEV^HYP^-mediated expansion of normal mammary gland stem and progenitor cells.

**Figure 3:**
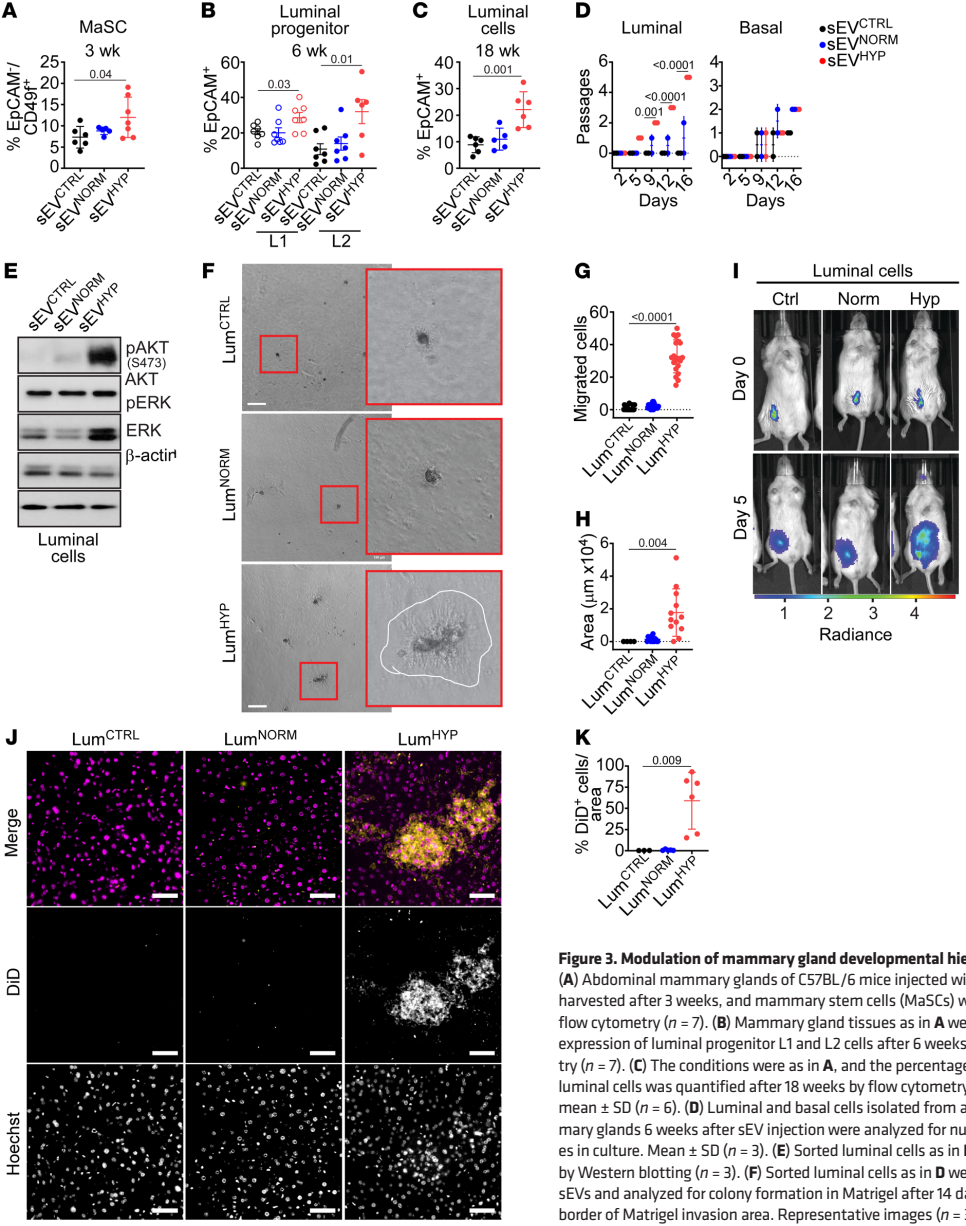
Modulation of mammary gland developmental hierarchy by sEV^HYP^. (**A**) Abdominal mammary glands of C57BL/6 mice injected with AT3 sEVs were harvested after 3 weeks, and mammary stem cells (MaSCs) were quantified by flow cytometry (*n* = 7). (**B**) Mammary gland tissues as in **A** were analyzed for expression of luminal progenitor L1 and L2 cells after 6 weeks by flow cytometry (*n* = 7). (**C**) The conditions were as in **A**, and the percentage of differentiated luminal cells was quantified after 18 weeks by flow cytometry. For panels **A**–**C**, mean ± SD (*n* = 6). (**D**) Luminal and basal cells isolated from abdominal mammary glands 6 weeks after sEV injection were analyzed for number of passages in culture. Mean ± SD (*n* = 3). (**E**) Sorted luminal cells as in **D** were analyzed by Western blotting (*n* = 3). (**F**) Sorted luminal cells as in **D** were treated with sEVs and analyzed for colony formation in Matrigel after 14 days. White line, border of Matrigel invasion area. Representative images (*n* = 3). Scale bars: 100 μm. (**G**) Sorted luminal cells as in **D** were analyzed for migration on PET inserts during 24 hours. Mean ± SD (*n* = 3). (**H**) The conditions were as in **F**, and the area of Matrigel invasion was quantified. Mean ± SD (*n* = 3). (**I**) Sorted luminal cells as in **D** were stained with Vybrant-DiD dye, injected in the mammary gland of immunocompromised NOD/SCID IL2Rγ^null^ mice, and tracked using an IVIS SpectrumCT In Vivo Imaging System at the time of injection and after 5 days. Representative images. (**J** and **K**) The conditions were as in **I**, and livers were analyzed after 8 weeks for DiD^+^ luminal cells by fluorescence microscopy (**J**, representative images) and quantified (**K**). Scale bars: 50 μm. Yellow, DiD^+^ cells; magenta, nuclei. Mean ± SD (*n* = 6). For all panels, numbers correspond to *P* values by 1-way ANOVA with Tukey’s multiple-comparison test.

To elucidate the mechanism(s) of sEV^HYP^-induced mammary gland atypia, we next examined potential changes in the hierarchy of epithelial differentiation. Injection of mouse mammary glands with AT3 cell–derived sEV^HYP^ significantly expanded a CD45^–^EpCAM^–^CD49^hi^CD24^lo^ cell population, corresponding to mammary stem cells (MaSCs), within 3 weeks of sEV exposure (*n* = 5 animals per group) (Figure 3A and Supplemental Table 2). This was accompanied by increased proliferation of mammary gland luminal 1 (L1, CD45^–^EpCAM^+^CD49f^+^CD24^hi^) and luminal 2 (L2, CD45^–^EpCAM^+^CD49f^+^) progenitor cells 6 weeks after sEV injection (*n* = 5 animals per group) (Figure 3B and Supplemental Table 2). These changes were specific, as EO771 cell–derived sEV^HYP^ did not increase the fraction of MaSCs (Supplemental Figure 3A and Supplemental Table 2) or L1 or L2 progenitor cells (Supplemental Figure 3B and Supplemental Table 2) after comparable time intervals in vivo (*n* = 4 animals per group). Consistent with these data, sEV^HYP^ expanded the population of differentiated CD45^–^EpCAM^+^CD49f^+^ mammary gland luminal cells 18 weeks after injection (*n* = 5 animals per group) (Figure 3C and Supplemental Table 2). Conversely, sEV^HYP^ did not affect the frequency of CD45^–^EpCAM^–^CD49f^+^ mammary gland basal cells at 3, 6, or 18 weeks after injection (Supplemental Figure 3C and Supplemental Table 2). In all experiments, injection with AT3 cell–derived sEV^NORM^ had no effect on the frequency of MaSCs (Figure 3A), L1 and L2 luminal progenitors (Figure 3B), differentiated luminal cells (Figure 3C), or basal cells (Supplemental Figure 3C) at comparable time intervals.

Next, we isolated luminal and basal cells from mammary glands injected with sEVs by fluorescence sorting (*n* = 6 animals per group). Consistent with increased Ki67 reactivity (Figure 1, C and D), treatment with sEV^HYP^ increased the proliferation of isolated luminal cells ex vivo (Figure 3D and Supplemental Figure 3D). This response was specific for luminal cells, as sEV^HYP^ did not affect the proliferation of mammary gland basal cells isolated under the same conditions (Figure 3D). Similar to the results obtained with HC11 cells (Figure 2G), sEV^HYP^ treatment of isolated luminal cells increased the phosphorylation of Akt and ERK ex vivo, whereas sEV^NORM^ had no effect (Figure 3E).

To rule out that the observed changes were unique to the cell lines used, we next examined an additional metastatic (4T1) and an additional non-metastatic (Brpkp110) mouse breast adenocarcinoma cell type. Normoxic or hypoxic sEVs isolated from 4T1 and Brpkp110 cells were comparable in yield and size distribution (Supplemental Figure 3E). When injected in the mammary fat pad of recipient female C57BL/6 mice, sEV^HYP^ but not sEV^NORM^ isolated from metastatic 4T1 cells induced extensive epithelial hyperplasia with increased Ki67 labeling (Supplemental Figure 3F) and prominent expansion of the EpCAM^–^CD49f^+^ MaSC compartment (Supplemental Figure 3G). In contrast, sEV^HYP^ or sEV^NORM^ isolated from non-metastatic Brpkp110 cells had no effect on the mammary epithelium (Supplemental Figure 3F) or MaSCs (Supplemental Figure 3G).

### Oncogenic changes in sEV^HYP^-treated mammary gland luminal cells.

Consistent with disrupted apical-basal polarity (Figure 1, F and G), isolated luminal cells exposed to sEV^HYP^ exhibited hallmarks of EMT, with increased levels of MMP-9, switching of N- and E-cadherin expression by Western blotting (Supplemental Figure 4A), and transcriptional upregulation of an EMT gene signature comprising LBX-1, Snail, Slug, Twist-1, and Nanog by reverse transcriptase PCR (RT-PCR) in vivo and ex vivo (Supplemental Figure 4B). sEV^NORM^ had no effect on EMT markers by Western blotting (Supplemental Figure 4A) or RT-PCR (Supplemental Figure 4B). In addition to EMT, sEV^HYP^-treated luminal cells exhibited increased colony formation in Matrigel (Figure 3F), resulting in larger surface area and reduced circularity of individual colonies (Supplemental Figure 4C), heightened migration on PET inserts (Figure 3G), and increased Matrigel invasion (Figure 3, F and H). In contrast, sEV^NORM^ had no effect on luminal cell colony formation, migration, or invasion in comparison with controls (Figure 3F and Supplemental Figure 4C).

To test whether sEV^HYP^ induced comparable oncogenic changes in vivo, luminal cells isolated from C57BL/6 mice treated with the various sEVs were labeled with DiD, injected in the mammary gland of recipient immunocompromised NOD/SCID IL2Rγ^null^ mice (*n* = 10 animals per group), and monitored for cell proliferation and local or systemic invasion by fluorescence imaging (Supplemental Figure 4D). Consistent with ex vivo data, exposure to sEV^HYP^ increased luminal cell proliferation in recipient mice compared with sEV^NORM^ or control animals (Figure 3I). In addition, luminal cells treated with sEV^HYP^ exhibited increased migration from the site of injection to occupy the entire mammary gland in vivo (Supplemental Figure 4E). This was accompanied by systemic dissemination of sEV^HYP^-stimulated luminal cells to the liver of recipient animals compared with controls (Figure 3, J and K, and Supplemental Figure 4F). This response showed organ tropism, since sEV^HYP^-exposed luminal cells did not disseminate to lungs, bone marrow (BM), brain, or spleen of reconstituted animals, by fluorescence analysis (Supplemental Figure 4F). In all experiments, exposure to sEV^NORM^ did not affect luminal cell migration in the mammary gland or dissemination to distant organs in vivo (Figure 3K and Supplemental Figure 4F).

### Hypoxic sEVs mediate systemic myeloid immunosuppression mediated by S100A9.

**Figure 4:**
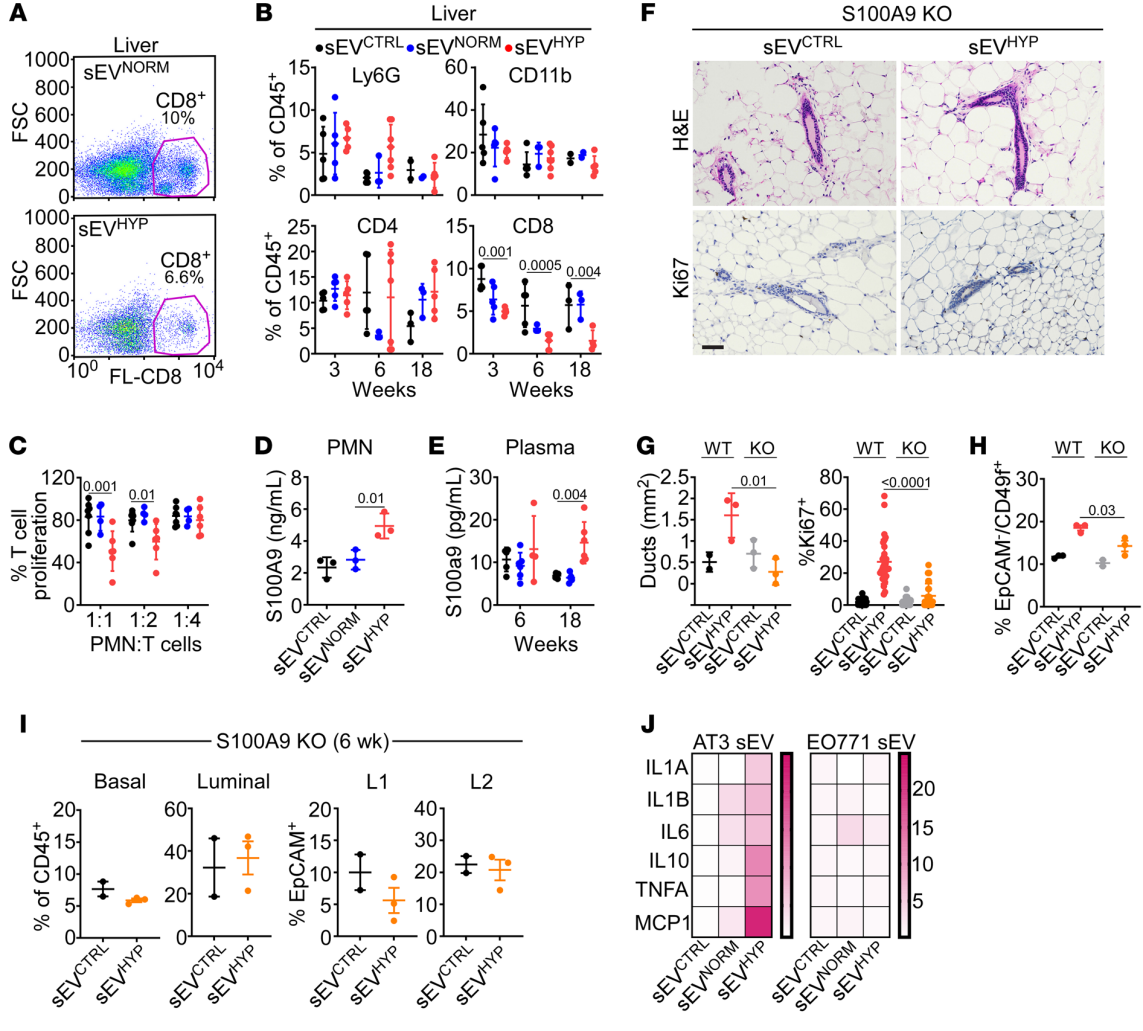
sEV^HYP^ promote myeloid cell immunosuppression and S100A9 release. (**A**) Liver samples from sEV^CTRL^- or sEV^HYP^-injected mice were analyzed for CD8^+^ T cell expression by flow cytometry. Representative experiment (*n* = 6). (**B**) Liver samples as in **A** were analyzed for myeloid (Ly6G^+^, CD11b^+^) or lymphoid (CD4^+^, CD8^+^) cell populations by flow cytometry. (**C**) T cells cocultured with naive mouse PMNs treated with AT3 cell–derived sEVs at the indicated ratios were analyzed for cell proliferation. Mean ± SD (*n* = 3). (**D**) Naive mouse PMNs were incubated with sEVs, and culture supernatants were analyzed for released S100A9 by ELISA (*n* = 3). (**E**) Plasma samples from sEV-injected mice were analyzed for S100A9 levels by ELISA (*n* = 5). (**F** and **G**) Wild-type (WT) or S100A9-knockout (KO) mice were injected in the mammary gland with sEV^CTRL^ or sEV^HYP^ and analyzed by IHC (**F**, representative images) with quantification of mammary duct expansion (**G**, left) and Ki67^+^ cell proliferation (**G**, right). Scale bar: 100 μm. Mean ± SD (*n* = 3). (**H**) The conditions were as in **F** and **G**, and mammary gland samples from WT or S100A9-KO mice were analyzed for MaSCs. Mean ± SD (*n* = 3). (**I**) Mammary gland tissues from S100A9-KO mice were analyzed for basal or luminal progenitor cells (left) or L1 and L2 differentiated luminal cells (right) 6 weeks after sEV injection. (**J**) PMNs incubated with sEVs isolated from AT3 (left) or EO771 (right) cells were analyzed for expression of the indicated cytokines by quantitative RT-PCR. Data are presented as heatmaps. Representative experiment (*n* = 3). For all panels, numbers correspond to *P* values by 1-way ANOVA with Tukey’s multiple-comparison test.

Based on the ability of sEV^HYP^-treated luminal cells to disseminate to the liver of recipient animals, we next asked whether sEV^HYP^ signaling induced systemic changes. Unexpectedly, C57BL/6 mice injected with sEV^HYP^ in the mammary gland exhibited a significant reduction in CD8^+^ T cell accumulation in the liver (Figure 4, A and B). In contrast, the fraction of myeloid Ly6G^+^ and CD11b^+^ cells or CD4^+^ T cells was not significantly affected (Figure 4B). These changes were specific, as immune profiling of the mammary gland (Supplemental Figure 5A) or spleen (Supplemental Figure 5B) of sEV^HYP^-injected mice did not show significant changes in the distribution of myeloid (Ly6G^+^, CD11b^+^) or lymphoid (CD4^+^, CD8^+^) cell populations. Similarly, sEV^HYP^ isolated from EO771 cells had no effect on myeloid or lymphoid cell populations in the liver of injected mice (Supplemental Figure 5C).

To examine a role of systemic immunity in sEV^HYP^ signaling, we next carried out additional reconstitution experiments in which luminal cells isolated from sEV^HYP^-treated mice were injected in the mammary gland of immunocompetent C57BL/6 animals (Supplemental Figure 5D) instead of immunocompromised NOD/SCID mice. In these experiments, reconstituted luminal cells failed to distribute throughout the mammary gland of C57BL/6 mice and did not localize to the liver (Supplemental Figure 5, E and F). Conversely, preconditioning of immunocompetent recipient mice with sEV^HYP^ 5 weeks before reconstitution (Supplemental Figure 5D) restored luminal cell infiltration of the mammary gland as well as accumulation in the liver (Supplemental Figure 5, E and F). Consistent with specific liver tropism, no accumulation of luminal cells was observed in the lung, BM, brain, or spleen of reconstituted animals with or without sEV^HYP^ preconditioning (Supplemental Figure 5G).

We next focused on myeloid cells as potential systemic effectors of sEV^HYP^-induced immune reprogramming. We found that PMNs isolated from sEV^HYP^-injected mice suppressed T cell proliferation in a concentration-dependent manner in a coculture assay (Figure 4C) and exhibited increased release of the alarmin and immunosuppressive mediator S100A9 (Figure 4D). This was accompanied by time-dependent increase in the plasma concentration of S100A9 in sEV^HYP^-injected mice (Figure 4E). Conversely, exposure to sEV^CTRL^ or sEV^NORM^ did not affect T cell proliferation or S100A9 production or circulating levels (Figure 4, C–E).

In addition to immunosuppression, S100A9 functions as a transcriptional coactivator during mammary gland oncogenic transformation (32), and we examined the role of this pathway in S100A9-knockout (S100A9-KO) mice. In these experiments, injection of S100A9-KO mice with sEV^HYP^ did not induce hyperplasia of mammary gland ducts (Figure 4F and Figure 4G, left) or increased Ki67-associated mammary epithelium proliferation (Figure 4F and Figure 4G, right) compared with the response of wild-type mice. In addition, S100A9-KO mice showed no expansion of MaSCs in response to sEV^HYP^ treatment (Figure 4H), and the fraction of basal or luminal progenitors as well as differentiated L1 and L2 populations was unchanged up to 6 weeks after injection, in comparison with sEV^CTRL^-injected mice (Figure 4I). A mechanistic basis for sEV^HYP^-induced PMN immunosuppression was next investigated. We found that AT3 cell–derived sEV^HYP^, but not sEV^NORM^, potently activated NF-κB–dependent gene expression in recipient PMNs, with upregulation of pleiotropic cytokines IL-1α, IL-1β, IL-6, IL-10, MCP-1, and TNF-α, which has been implicated in promoting the expansion (33) and immunosuppressive properties of myeloid-derived suppressor cells in vivo (34) (Figure 4J). Conversely, sEVs from normoxic or hypoxic EO771 cells did not induce changes in cytokine expression, including TNF-α (Figure 4J).

### Stimulation of mammary gland angiogenesis by sEV^HYP^.

**Figure 5:**
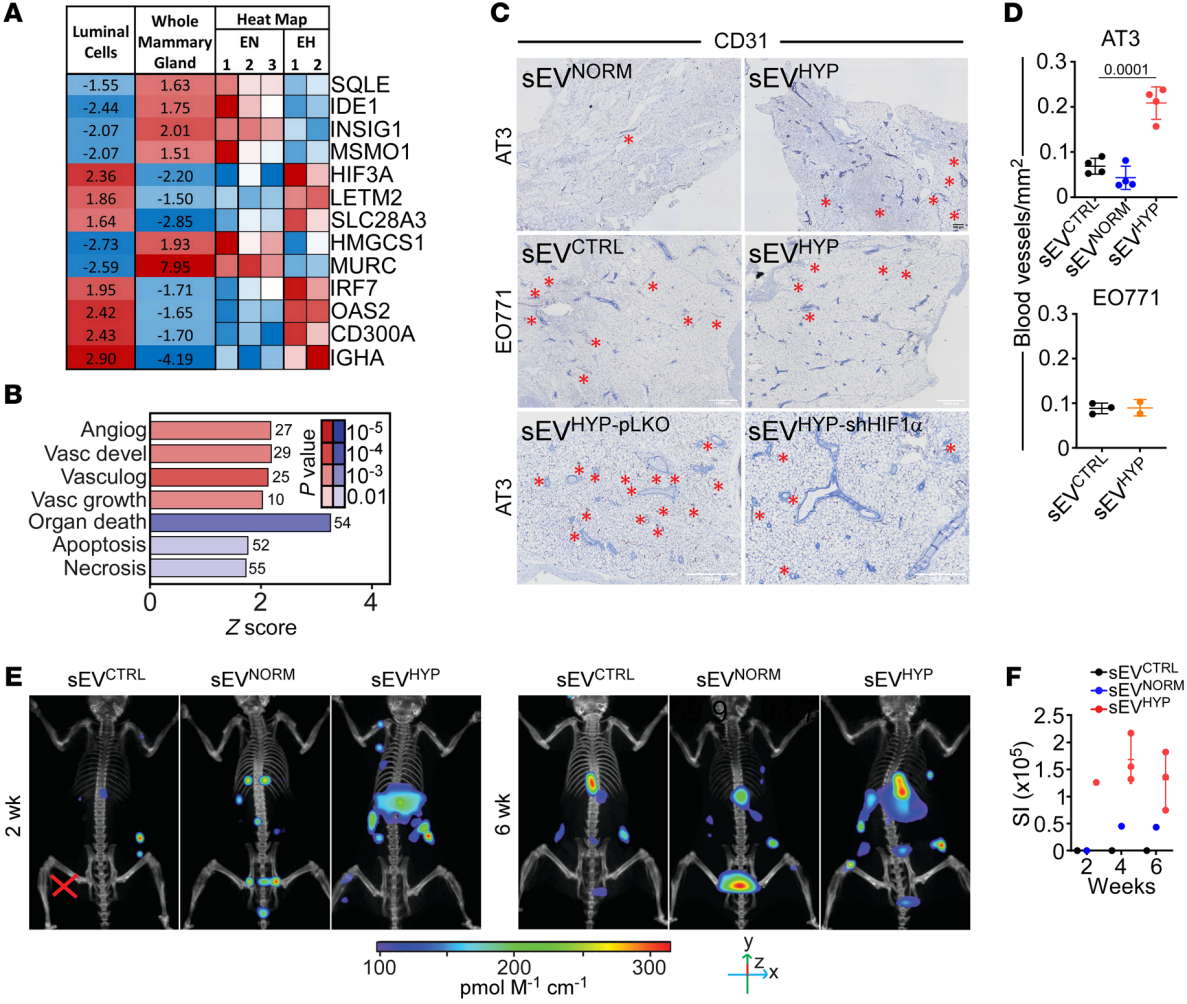
sEV^HYP^ regulation of mammary gland angiogenesis. (**A**) Whole mammary glands or isolated luminal cells treated with sEV^NORM^ (EN) or sEV^HYP^ (EH) were analyzed for changes in gene expression by RNA-Seq. Data are expressed as a heatmap. Mean expression changes in EH/EN comparisons as well as individual replicates of luminal cell expression levels versus mean are indicated. Blue, downregulated; red, upregulated. (**B**) The conditions were as in **A**, and pathways activated (red) or inhibited (blue) in isolated luminal cells treated with sEV^HYP^ were quantified. The number of genes and *P* values are indicated. FDR < 10%; *z* score ≥ 1.5. (**C**) AT3 or EO771 cell–derived sEVs were injected in the abdominal mammary gland of C57BL/6 mice, and tissue samples were analyzed for expression of CD31 after 6 weeks by IHC (representative images). Black scale bar: 500 μm; white scale bars: 1,000 μm. Red asterisks, blood vessels. Top: AT3 cell–derived sEV^NORM^ or sEV^HYP^. Middle: EO771 cell–derived sEV^NORM^ or sEV^HYP^. Bottom: AT3 cell–derived sEV^pLKO^ or sEV^shHIF1α^. (**D**) The conditions were as in **C**, and microvessel density was quantified from CD31 reactivity by IHC. Mean ± SD (*n* = 4). Numbers correspond to *P* values by 1-way ANOVA with Tukey’s multiple-comparison test. (**E**) AT3 cell–derived sEVs were injected in the abdominal mammary gland of C57BL/6 mice followed by i.v. administration of IVISense Vascular 750 Fluorescent Probe IV after 2 (left) and 6 (right) weeks (*n* = 3). (**F**) The conditions were as in **E**, and the IVISense fluorescence signal was quantified after 24 hours by CT scan. SI, source intensity.

Based on these findings, we next profiled the transcriptome of isolated luminal cells treated with sEV^HYP^ by RNA-Seq (*n* = 2–3 animals per group). Compared with sEV^NORM^, exposure of luminal cells to sEV^HYP^ increased the expression of genes (top fold changes [FCs]) implicated in cell-cell contact and cytoskeletal rearrangement (Cd248, FC = 6, *P* = 0.03; Efhd1, FC = 4.5, *P* = 0.01; Syd1, FC = 3.1, *P* = 0.02; Mfap4, FC = 2.7, *P* = 0.01), EMT (Fhl3, FC = 3.6, *P* = 0.003), bioenergetics (Pygm, FC = 2.9, *P* = 0.01; Chac1, FC = 2.9, *P* = 0.01; Cacnb1, FC = 1.9, *P* = 0.03), and inflammation/innate immunity (Ccl27, FC = 2.4, *P* = 0.003; Oas2, FC = 2.4, *P* = 0.03; Cd70, FC = 2.4, *P* = 0.03). Conversely, genes involved in cell death (Aifm3, FC = –1.9, *P* = 0.04) or tumor suppression (SerpinB2, FC = –35.0, *P* = 7 × 10^–4^; Arhgap15, FC = –3.6, *P* = 0.03; Frk, FC = –1.7, *P* = 0.01) were downregulated. Certain changes were specific for luminal cells, as cholesterol biosynthesis that was increased in mammary glands injected with sEV^HYP^ was downregulated in isolated luminal cells (Hmgcr, FC = –1.7, *P* = 0.03; Hmgcs1, FC = –2.7, *P* = 0.008; Idi1, FC = –2.4, *P* = 0.006) (Figure 5A).

In addition, sEV^HYP^ stimulation of luminal cells increased the expression of genes implicated in angiogenesis and vasculogenesis, compared with sEV^NORM^ or sEV^CTRL^ (Egr1, FC = 1.9, *P* = 0.002; Egfl6, FC = 3, *P* = 0.03; Dab2, FC = 1.7, *P* = 0.04; Ndp, FC = 2.9, *P* = 0.02; Ntn4, FC = 1.9, *P* = 0.01; Sema3c, FC = 1.8, *P* = 0.01) (Figure 5B). Consistent with this prediction, AT3 cell–derived sEV^HYP^ potently increased new blood vessel formation in recipient mammary glands by CD31 staining and immunohistochemistry in vivo (Figure 5, C and D, top). In contrast, EO771 cell–derived sEV^HYP^ had no effect on angiogenesis in vivo (Figure 5C, middle, and Figure 5D, bottom). As an independent approach, we visualized angiogenesis in live mice by fluorescence imaging. Consistent with the data above, sEV^HYP^ treatment induced extensive angiogenesis in the mammary gland as early as 2 weeks after injection and persisting throughout 6 weeks of observation (Figure 5, E and F). Instead, sEV^NORM^ did not stimulate angiogenesis in recipient mice compared with controls (Figure 5, E and F).

### Clathrin-dependent accumulation of HIF1α in sEV^HYP^.

HIF1α is a central regulator of angiogenesis, and its role in sEV^HYP^-induced mammary gland blood vessel formation was next examined. We found that metastatic breast adenocarcinoma AT3 and 4T1 cells exposed to hypoxia expressed HIF1α protein (Supplemental Figure 6A) and mRNA (Supplemental Figure 6B) in their sEV^HYP^. In contrast, sEV^HYP^ of non-metastatic EO771 and Brpkp110 cells were devoid of HIF1α protein (Supplemental Figure 6C) and mRNA (Supplemental Figure 6D). In addition, sEV^NORM^ from metastatic cells were also negative for HIF1α protein and mRNA (Supplemental Figure 6, A and B), suggesting that exposure to hypoxia promotes the packaging of HIF1α into sEV^HYP^. To elucidate the mechanistic requirements of this pathway, we next used 2 independent siRNA sequences to silence the expression of clathrin, a key effector of endocytic vesicle assembly, in AT3 cells (Supplemental Figure 6E). Here, depletion of clathrin (Supplemental Figure 6E) did not affect overall sEV size (Supplemental Figure 6F) whereas sEVs yield was significantly reduced, in comparison with control transfectants (Supplemental Figure 6G). Under these conditions, clathrin silencing strongly reduced HIF1α accumulation in hypoxic sEVs (Supplemental Figure 6, E and H).

### sEV^HYP^-HIF1α regulation of mammary gland angiogenesis.

**Figure 6:**
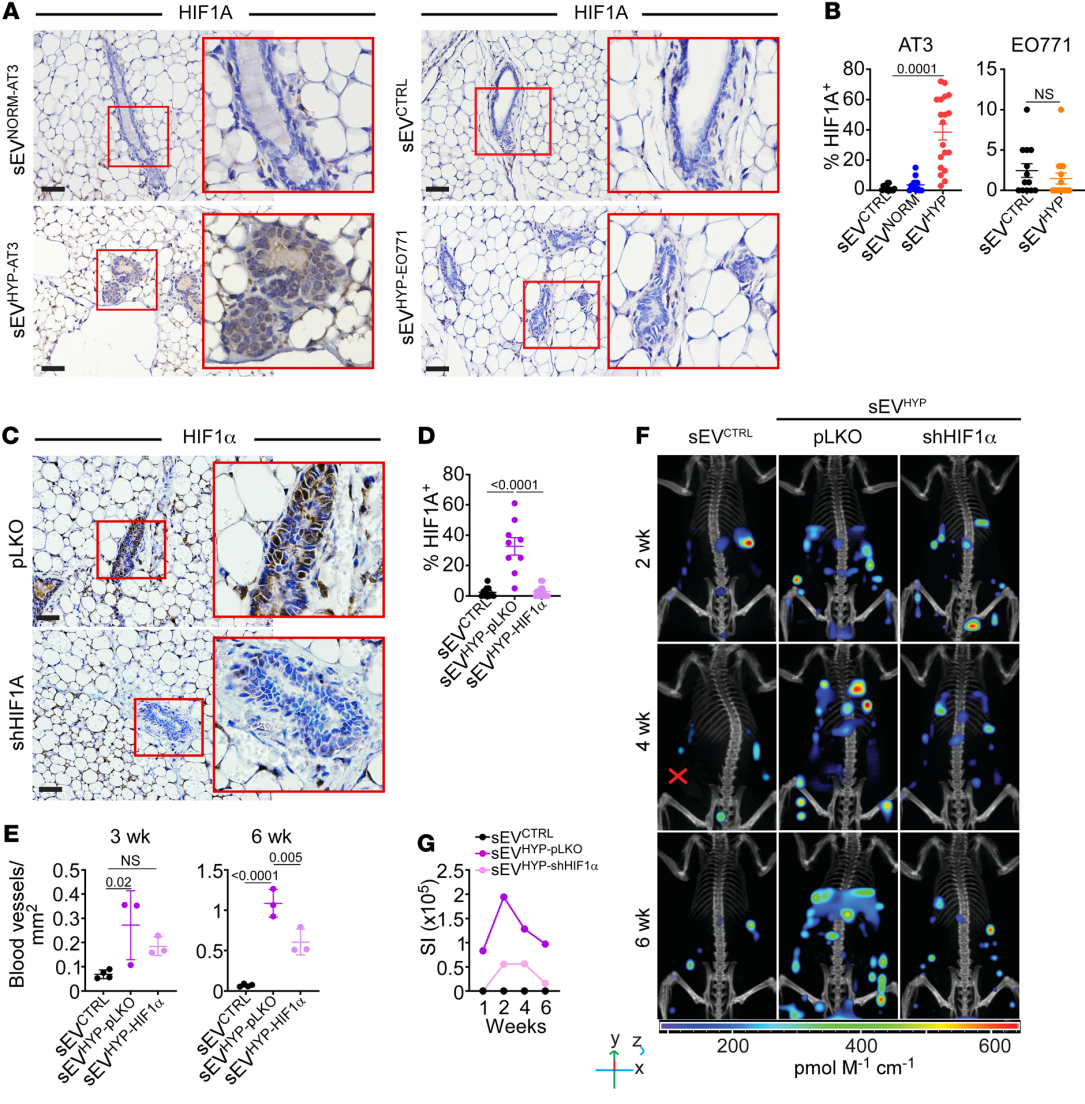
sEV^HYP^-HIF1α signaling controls mammary gland angiogenesis. (**A** and **B**) AT3 or EO771 cell–derived sEVs were injected in the abdominal mammary gland of C57BL/6 mice, and tissue samples were analyzed for nuclear expression of HIF1α after 6 weeks by IHC (**A**, representative images) and quantified (**B**). Scale bars: 100 μm. Mean ± SD (*n* = 4). (**C** and **D**) sEV^HYP^ isolated from AT3 cells transduced with pLKO or shHIF1α were injected in the abdominal mammary gland of C57BL/6 mice, and tissue samples were analyzed for HIF1α expression after 6 weeks by IHC (**C**, representative images) and quantified (**D**). Red boxes, magnification of indicated areas. Scale bars: 100 μm. Mean ± SD (*n* = 3). (**E**) Mammary glands injected with AT3 cell–derived sEV^pLKO^ or sEV^shHIF1α^ were analyzed for microvessel density by CD31 staining and IHC. Mean ± SD (*n* = 3). (**F** and **G**) The conditions were as in **C** and **D**, and mice injected i.v. with IVISense Vascular 750 Fluorescent Probe after 2, 4, and 6 weeks were analyzed after an additional 24 hours by CT scan on an IVIS Spectrum (**F**, representative 3D reconstructed images) with quantification of fluorescence intensity (**G**) (*n* = 3). For all panels, numbers correspond to *P* values by 1-way ANOVA with Tukey’s multiple-comparison test.

Consistent with their HIF1α content, AT3 cell–derived sEV^HYP^ upregulated HIF1α mRNA (Supplemental Figure 7A) and protein (Supplemental Figure 7B) expression in isolated mammary gland luminal cells, by RT-PCR and Western blotting, respectively. Expression of hydroxylated, i.e., degradable HIF1α was undetectable in these conditions (Supplemental Figure 7B), consistent with HIF1α stabilization. Similarly, a member of the proly-hydroxylase family implicated in HIF1α degradation, PHD-2, was also undetectable after sEV^HYP^ treatment (Supplemental Figure 7B). In contrast, AT3 cell–derived sEV^NORM^, which lack HIF1α, had no effect on HIF1α mRNA or protein expression in isolated luminal cells (Supplemental Figure 7, A and B). Similar results were observed in vivo, as AT3 cell–derived sEV^HYP^ upregulated HIF1α levels in the mammary glands of C57BL/6 mice by immunohistochemistry (Figure 6, A and B), whereas EO771 cell–derived sEV^HYP^ or sEV^NORM^ from AT3 cells had no effect (Figure 6, A and B). The increased accumulation of HIF1α induced by sEV^HYP^ was long-lasting, as analysis of the mammary epithelium up to 18 weeks after sEV exposure exhibited increased HIF1α expression by immunohistochemistry (Supplemental Figure 7, C and D) and Western blotting on isolated luminal cells, concomitantly with loss of PHD-2 expression (Supplemental Figure 7E).

To test whether HIF1α vehiculated by sEV^HYP^ contributed to mammary gland angiogenesis, we next established clones of AT3 cells stably transduced with control pLKO or 2 independent HIF1α-directed shRNA sequences (Supplemental Figure 7F). These cells lacked detectable HIF1α in whole cell extracts and produced sEVs by Western blotting (Supplemental Figure 7F). In control experiments, the yield and size distribution of HIF1α-depleted sEVs were comparable to those in control pLKO-transduced cultures (Supplemental Figure 7G). Consistent with the data above, sEV^HYP^ from pLKO cultures increased the expression of HIF1α in the mammary gland epithelium in vivo (*n* = 3 animals per group) (Figure 6, C and D). In contrast, HIF1α-depleted sEV^HYP^ had no effect on HIF1α levels (Figure 6, C and D) and did not stimulate mammary gland angiogenesis throughout a 3-week and a 6-week interval by immunohistochemistry (Figure 5C, bottom, and Figure 6E). Similar results were obtained by whole-animal live fluorescence imaging. Here, sEV^HYP^ from control pLKO clones stimulated increased angiogenesis starting at 2 weeks after injection and throughout a 6-week observation period (Figure 6, F and G). In contrast, shHIF1α silencing abolished angiogenesis at all time points examined in vivo (Figure 6, F and G).

### Requirement of HIF1α for sEV^HYP^-induced luminal cell expansion and S100A9 signaling.

**Figure 7:**
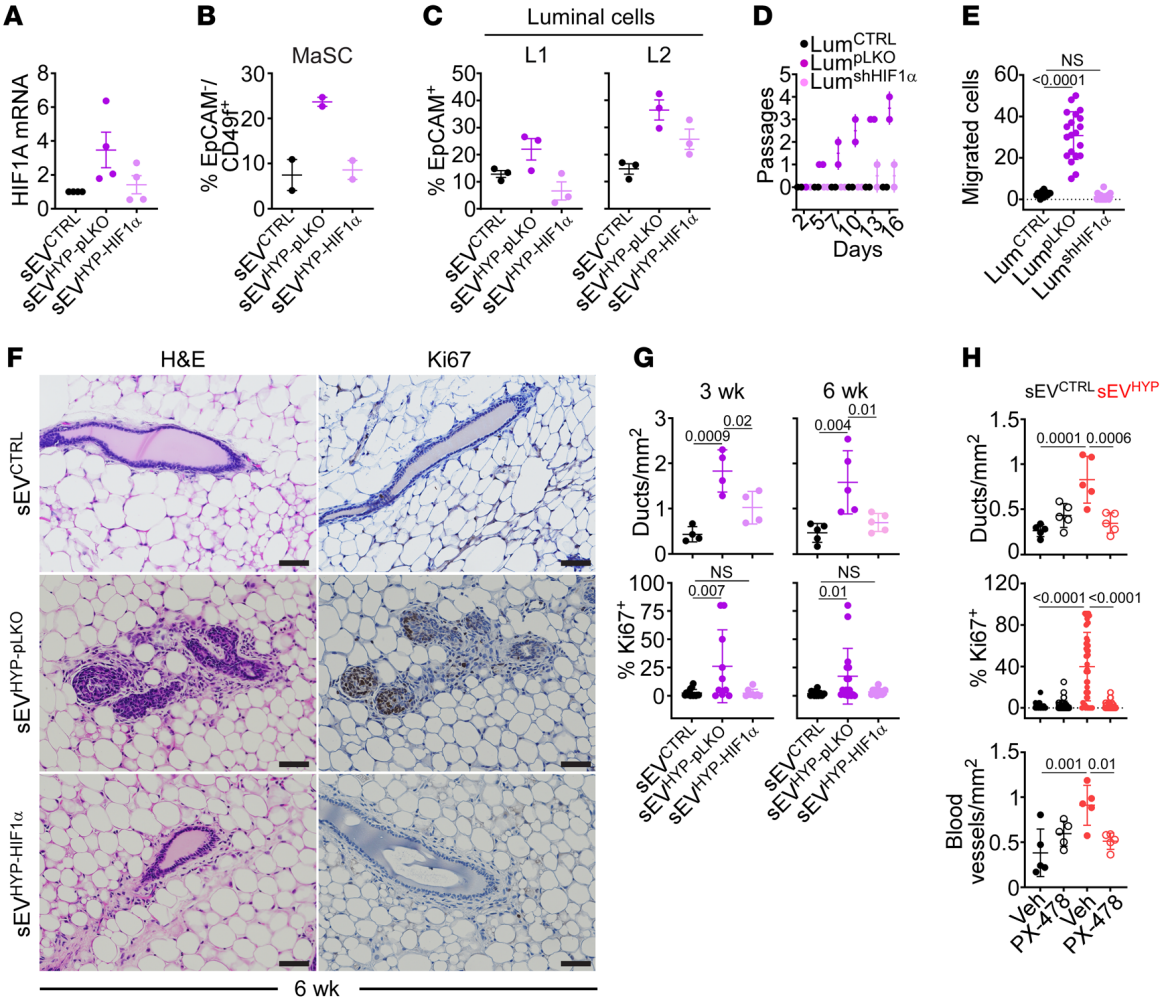
sEV^HYP^-HIF1α modulation of mammary gland developmental hierarchy. (**A**) sEV^HYP^ from AT3 cells transduced with pLKO (sEV^pLKO^) or shHIF1α (sEV^shHIF1α^) were injected in the abdominal mammary gland of C57BL/6 mice, and luminal cells isolated after 6 weeks were quantified for HIF1α mRNA expression by quantitative RT-PCR. Mean ± SD (*n* = 4). (**B** and **C**) The conditions were as in **A**, and the percentage of mammary stem cells (MaSC, *n* = 2) (**B**) or luminal L1 and L2 progenitor cells (**C**, *n* = 3) was quantified after 3 (**B**) or 6 (**C**) weeks by flow cytometry. (**D**) Luminal cells were sorted from the mammary gland of C57BL/6 mice as in **A** and analyzed for the number of passages in culture (*n* = 3). (**E**) The conditions were as in **D**, and migration of sorted luminal cells on PET inserts was quantified. Mean ± SD (*n* = 3). (**F** and **G**) Mammary glands as in **A** were analyzed after 3 and 6 weeks from sEV injection (**F**, representative images at 6 weeks, *n* = 5) with quantification of mammary ducts and percentage of Ki67^+^ cells (**G**) by IHC. Scale bars: 100 μm. Mean ± SD. (**H**) C57BL/6 mice injected with sEV^CTRL^ or sEV^HYP^ in the abdominal mammary gland were given the small-molecule HIF1α inhibitor PX-478 on days 1 and 3 and analyzed after 21 days for mammary ducts (top), percentage of Ki67^+^ cells (middle), or blood vessel density (bottom) by IHC. Mean ± SD (*n* = 5). For all panels, numbers correspond to *P* values by 1-way ANOVA with Tukey’s multiple-comparison test.

Next, we asked whether the effect of sEV^HYP^-vehiculated HIF1α extended beyond angiogenesis and influenced mammary gland differentiation and systemic immunosuppression. Consistent with the results in mammary glands, HIF1α-depleted sEV^HYP^ had no effect on *HIF1A* mRNA levels in isolated luminal cells, compared with pLKO controls, by RT-PCR (Figure 7A). Under these conditions, depletion of HIF1α suppressed the ability of sEV^HYP^ to expand MaSCs (*n* = 2 animals per group) (Figure 7B and Supplemental Table 2), as well as L1 and L2 progenitors (*n* = 3 animals per group) (Figure 7C and Supplemental Table 2) in mammary glands, in vivo. In line with the results above, sEV^HYP^ from control pLKO-transduced cells increased the fraction of both MaSCs and L1 and L2 progenitors in vivo (Figure 7, B and C, and Supplemental Table 2). Functionally, silencing of HIF1α in AT3 cell–derived sEV^HYP^ abolished proliferation (Figure 7D) and migration (Figure 7E) of isolated luminal cells, in comparison with sEV^HYP^ from pLKO-transduced AT3 cells, ex vivo. In addition, HIF1α depletion reversed the increase in circulating S100A9 levels in response to sEV^HYP^ treatment (Supplemental Figure 8A). This was associated with suppression of NF-κB gene expression and cytokine induction in recipient PMNs (Supplemental Figure 8B) and normalization of T cell proliferation in coculture experiments with isolated PMNs (Supplemental Figure 8C).

In terms of biochemical markers, depletion of HIF1α from sEV^HYP^ prevented the emergence of EMT in isolated luminal cells by RT-PCR (Supplemental Figure 8D) and suppressed the increase in Akt or ERK phosphorylation compared with controls (Supplemental Figure 8E). Consistent with the data above, sEV^HYP^ from control pLKO-transduced AT3 cells induced EMT and stimulated Akt and ERK phosphorylation in isolated luminal cells (Supplemental Figure 8, D and E). Finally, injection of HIF1α-depleted sEV^HYP^ in the mammary gland of C57BL/6 mice did not induce mammary gland hyperplasia, new mammary duct formation, or increased Ki67^+^ cell proliferation, compared with control pLKO-sEV^HYP^, by immunohistochemistry (Figure 7, F and G).

As an independent experimental approach, we next treated C57BL/6 mice reconstituted with AT3 cell–derived sEV^CTRL^ or sEV^HYP^ with PX-478 (*n* = 5 animals per group), a melphalan-derived small-molecule HIF1α inhibitor currently in clinical development as an anticancer agent. Treatment with PX-478 or vehicle on day 1 and day 3 after sEV^CTRL^ or sEV^HYP^ injection was well tolerated with no changes in animal body weight throughout a 3-week observation period (Supplemental Figure 8F and Supplemental Table 1). Under these conditions, administration of PX-478 abolished HIF1α upregulation in the mammary gland mediated by sEV^HYP^, whereas vehicle had no effect (Supplemental Figure 8, G and H). Similar to the results obtained with HIF1α shRNA silencing, administration of PX-478 suppressed sEV^HYP^-induced mammary duct hyperplasia, abolished Ki67-associated cell proliferation, and prevented CD31-associated angiogenesis in vivo (Figure 7H and Supplemental Figure 8I). Conversely, PX-478 had no effect on mammary duct formation, epithelial cell proliferation, and angiogenesis in mice injected with sEV^CTRL^ (Figure 7H and Supplemental Figure 8I).

### sEV^HYP^-HIF1α signaling accelerates breast tumorigenesis.

**Figure 8:**
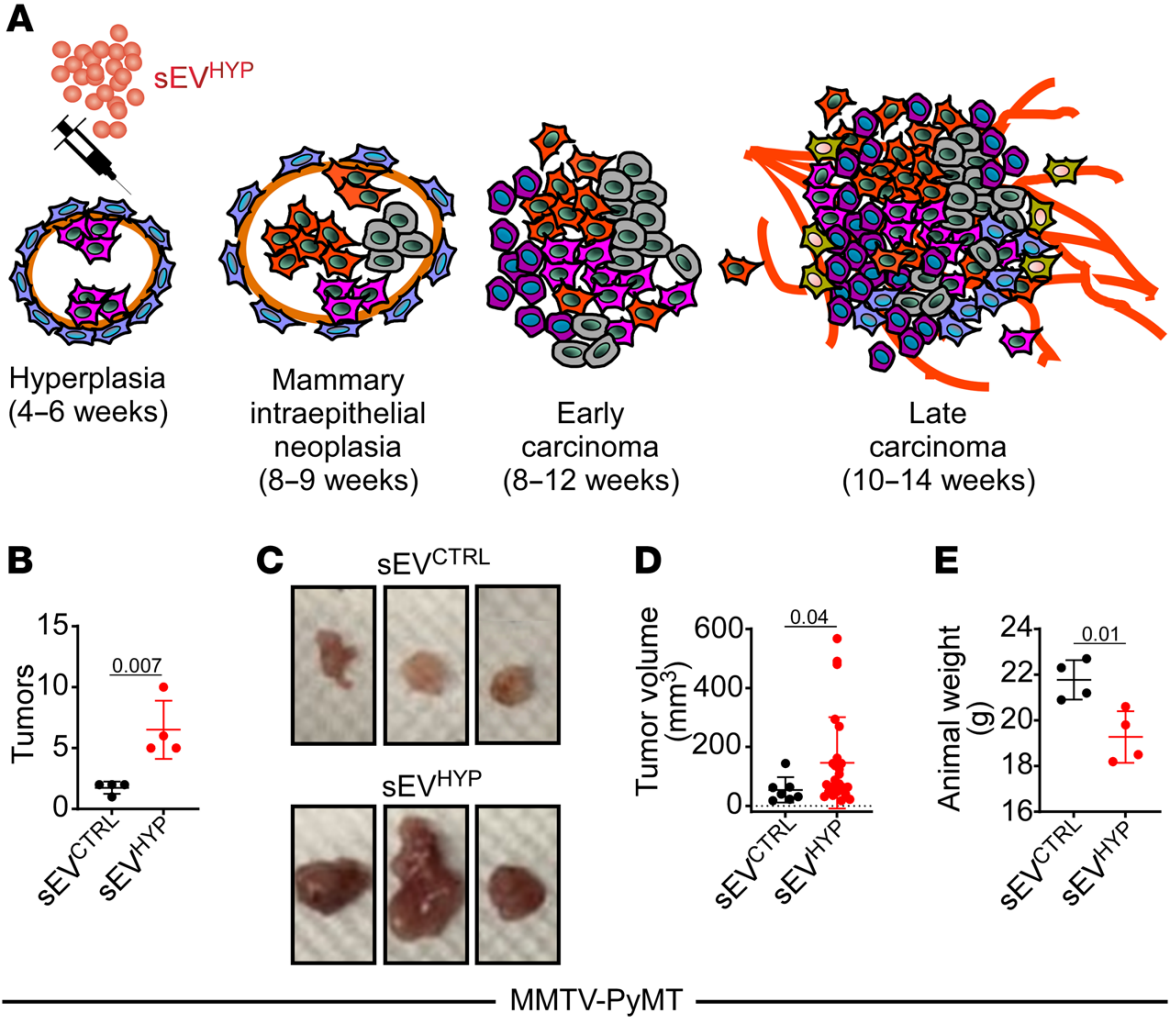
sEV^HYP^-HIF1α signaling accelerates breast tumorigenesis in vivo. (**A**) Schematic diagram of timeline of mammary gland tumorigenesis and disease progression in MMTV-PyMT–transgenic mice. (**B**) MMTV-PyMT mice (6 weeks old) were injected in the abdominal mammary gland with sEV^CTRL^ or AT3 cell–derived sEV^HYP^ and examined for differential tumor formation after 3 weeks. (**C** and **D**) Representative macroscopic images of tumors formed in MMTV-PyMT–transgenic mice after sEV injection (**C**) and quantification of tumor volume (**D**). (**E**) Weight of sEV-injected MMTV-PyMT–transgenic mice. For all panels, data are the mean ± SD (*n* = 4). Numbers correspond to *P* values by 1-way ANOVA with Tukey’s multiple-comparison test.

The data above suggest that sEV^HYP^ activate multiple transcriptional, metabolic, and signaling pathways that result in mammary gland hyperplasia/dysplasia. To test whether these changes accelerate full-blown breast tumorigenesis in vivo, we next injected sEVs from hypoxic AT3 cells in the mammary fat pad of MMTV-PyMT mice. This is an established model of human breast cancer progression, where expression of the polyoma virus middle T antigen (PyMT) under the control of mouse mammary tumor virus promoter/enhancer (MMTV) sequences results in mammary intraepithelial neoplasia by 9 weeks, early mammary gland adenocarcinoma by 12 weeks, and full-blown adenocarcinoma by 14 weeks (Figure 8A). In these experiments, 6-week-old MMTV-PyMT mice were injected with control or sEV^HYP^ in the mammary fat pad, and tumors were harvested after an additional 3-week time interval. Injection of sEV^HYP^ in these settings dramatically accelerated bilateral breast cancer progression in MMTV-PyMT mice, resulting in increased number and size of mammary tumors at 9 weeks of age, in comparison with MMTV-PyMT mice injected with sEV^CTRL^ (Figure 8, B–D). Accordingly, sEV^HYP^ treatment was associated with systemic animal weight loss after the same time interval, in comparison with control mice (Figure 8E).

### Role of sEV^HYP^-HIF1α signaling in luminal breast cancer recurrence.

**Figure 9:**
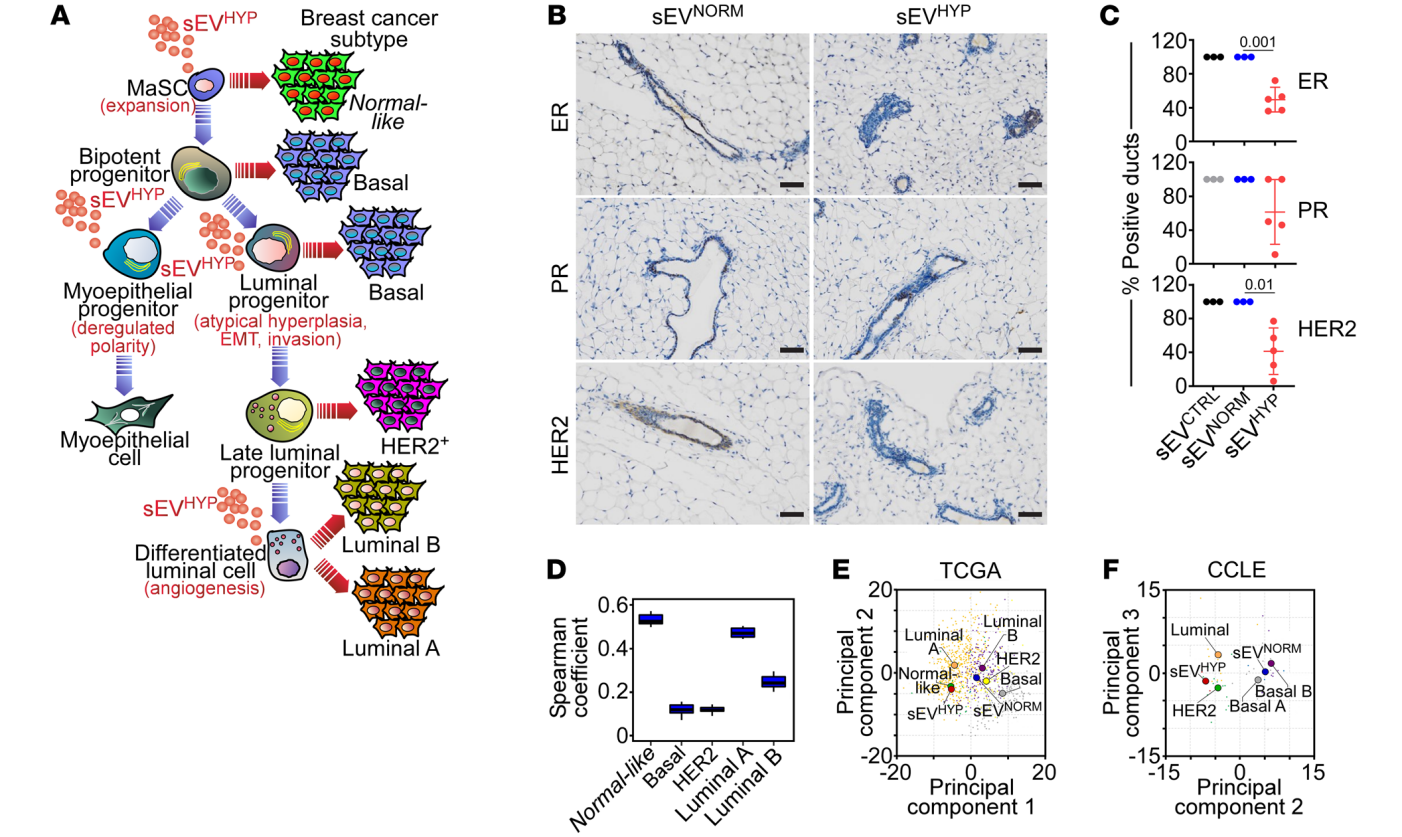
sEV^HYP^ modulation of luminal breast cancer development. (**A**) Schematic diagram of hierarchy of mammary epithelial cell differentiation and proposed origin of breast cancer subtypes. The multiple differentiation stages affected by sEV^HYP^ and resulting cellular responses are indicated in red. (**B** and **C**) sEV-injected mammary glands from C57BL/6 mice were analyzed for hormone receptor ER or PR status and HER2 expression by IHC (**B**, representative images) and quantified (**C**). Scale bars: 100 μm. Mean ± SD. Numbers correspond to *P* values by 1-way ANOVA with Tukey’s multiple-comparison test. (**D**) RNA-Seq data of luminal cells exposed to sEV^HYP^ in vivo were correlated with a breast cancer TCGA data set using a 50-gene PAM50 signature for breast cancer subtyping. The correlation with the individual intrinsic breast cancer subtypes is indicated. (**E** and **F**) Relationship between expression levels of genes modulated by sEV^HYP^ versus sEV^NORM^ and breast cancer subtypes in the TCGA (**E**) and CCLE (**F**) databases.

The data presented above suggest that sEV^HYP^-HIF1α signaling disrupts multiple steps in the hierarchy of mammary gland differentiation with expansion of MaSCs and L1 and L2 luminal progenitors, deregulated expression and localization of myoepithelial markers, and increased luminal cell proliferation, EMT, and local and distant luminal cell invasion (Figure 9A). Collectively, this pathway drives the onset and progression of high-risk breast premalignant lesions that predispose to full-blown breast adenocarcinomas when a driving oncogene is present (Figure 8, A–E).

To further explore a link between mammary gland lesions induced by sEV^HYP^ and human breast cancer, we first examined the expression of HER2 and hormone receptors ER and PR in these settings. Injection of mammary glands with sEV^HYP^, but not sEV^NORM^, caused heterogeneous and overall reduced ER and PR expression in comparison with controls by immunohistochemistry (Figure 9, B and C). Decreased HER2 levels were also observed in response to sEV^HYP^, compared with sEV^NORM^ (Figure 9, B and C). In addition, bioinformatics analysis of the PAM50 gene signature suggested that the transcriptome induced by sEV^HYP^ in luminal cells resembled the gene expression profile of normal-like breast cancer and luminal A breast cancer (Figure 9D). Similar results were obtained with analysis of the breast cancer subset of The Cancer Genome Atlas (TCGA) database, where the transcriptome of sEV^HYP^ mammary gland lesions more closely aligned with normal-like breast cancer and luminal breast cancer (Figure 9E). With the limitations of mouse-to-human comparison, inspection of human breast cancer cell lines in the Cancer Cell Line Encyclopedia (CCLE) also showed that the sEV^HYP^-induced transcriptome preferentially aligned with luminal breast cancer cell types, although similarities were also found with HER2^+^ cell lines (Figure 9F). Finally, whole genome sequencing analysis of luminal cells isolated 6 weeks after sEV^HYP^ injection in vivo showed no significant increase in the number of mutations (Supplemental Figure 9A), including coding mutations (Supplemental Figure 9B) or alterations in key breast cancer genes, such as TP53, PIK3CA, MYC, PTEN, CCND1, ERBB2, FGFRI, or GATA3, in comparison with controls.

**Figure 10:**
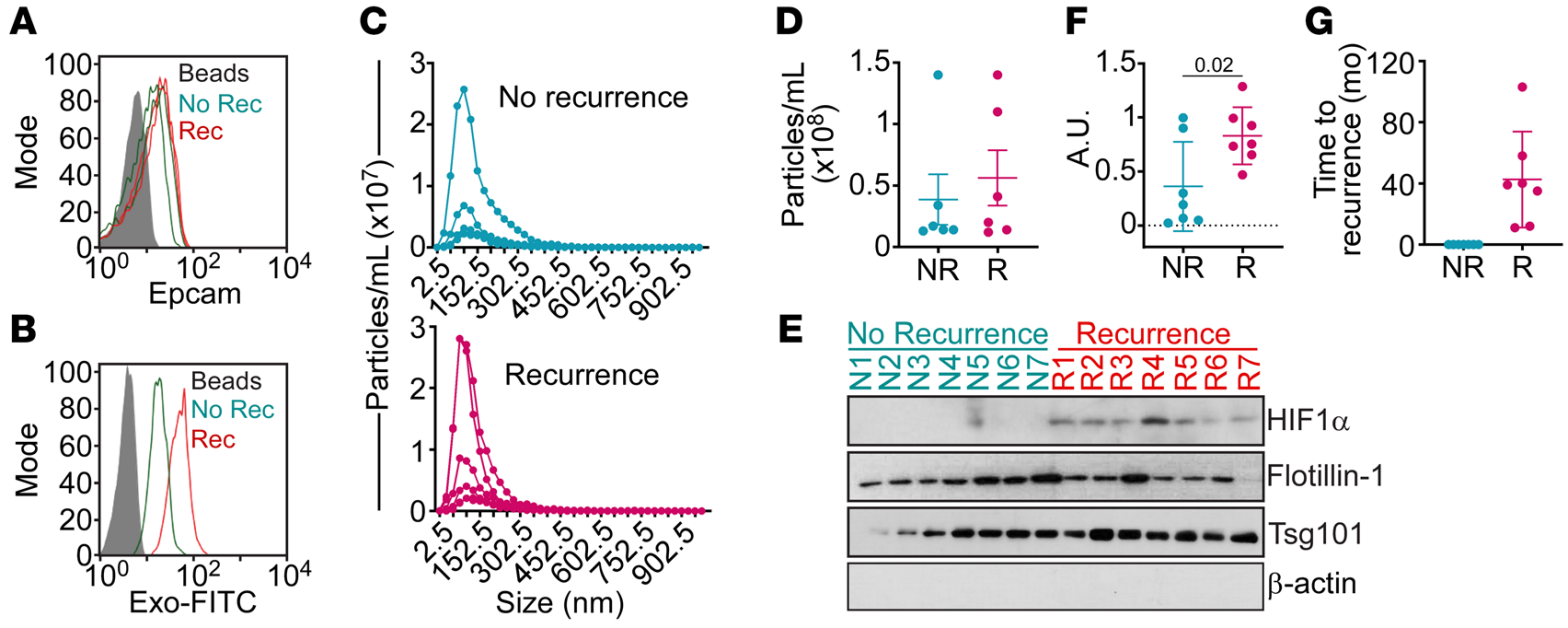
sEV-packaged HIF1α as a plasma biomarker of recurrent luminal breast cancer. (**A**) Circulating sEVs isolated from plasma of patients with recurrent (Rec) or non-recurrent (No Rec) breast cancer were analyzed for EpCAM expression using CD63^+^ beads by flow cytometry. Representative experiment. (**B**) The conditions were as in **A**, and plasma circulating sEVs were further enriched using EpCAM^+^ beads. sEV binding to beads was confirmed by Exo-FITC staining and flow cytometry. Representative experiment. (**C**) Circulating sEVs from patients with luminal breast cancer with or without recurrence (*n* = 14) were analyzed using a ZetaView analyzer with quantification of sEV number and size distribution. (**D**) Yield of circulating sEVs isolated from patients with luminal breast cancer with (R) or without (NR) recurrence. Each symbol corresponds to an individual patient. (**E** and **F**) sEVs isolated from plasma of patients with recurrent (*n* = 7) or non-recurrent (*n* = 7) breast cancer were analyzed by Western blotting (**E**), and protein bands were quantified by densitometry (**F**). Each symbol corresponds to an individual patient. AU, arbitrary units. Mean ± SD. Numbers correspond to *P* values by 1-way ANOVA with Tukey’s multiple-comparison test. (**G**) Time to recurrence (mo) of patients (*n* = 14) with diagnosis of recurrent or non-recurrent luminal breast cancer. Each symbol corresponds to an individual patient.

Based on these findings, we next examined a potential correlation between sEV^HYP^-HIF1α signaling and luminal breast cancer progression. For these studies, we developed a protocol to enrich and isolate plasma circulating sEVs from patients with confirmed diagnosis of luminal breast cancer with or without clinical recurrence (Supplemental Table 3). The circulating sEV population from these patients was isolated by size exclusion chromatography, followed by analysis of EpCAM expression by flow cytometry (Figure 10A) and enrichment of breast cancer–derived sEVs using EpCAM^+^ beads (Figure 10B). The resulting sEV samples showed comparable size distribution (Figure 10C) and yield (Figure 10D), irrespective of clinical recurrence or non-recurrence. Under these conditions, detection of HIF1α in plasma circulating sEVs was feasible by Western blotting and correlated with clinical recurrence in all luminal breast cancer patients examined (Figure 10, E and F), up to 103 months after initial diagnosis (Figure 10G). In contrast, luminal breast cancer patients without recurrence had no detectable HIF1α in plasma circulating sEVs (Figure 10, E and F).

## Discussion

In this study, we have shown that sEVs released by hypoxic breast cancer cells (sEV^HYP^) induce a comprehensive reprogramming of the normal mammary epithelium that involves both local and systemic changes. Locally, this pathway disrupts multiple steps in the differentiation hierarchy of the mammary gland epithelium, expanding MaSCs and L1 and L2 luminal progenitors, while inducing EMT and unbalanced apical-basal myoepithelial polarity. This is accompanied by oncogenic traits of sustained angiogenesis, resistance to cell death, and local and distant luminal cell invasion. At the systemic level, sEV^HYP^ signaling promotes myeloid immunosuppression and increased release of a pleiotropic “alarmin,” S100A9, which promotes both immune evasion and mammary gland transformation. As a result, sEV^HYP^ induce a unique mammary gland phenotype with atypical hyperplasia, intraepithelial neoplasia, and deregulated hormone and HER2 receptor expression with global transcriptional changes that resemble luminal breast cancer. Against the genetic backdrop of a well-established mammary gland driver oncogene (35), this pathway is sufficient to accelerate bilateral breast cancer onset and progression. Mechanistically, both local and systemic aspects of sEV^HYP^ signaling are mediated by HIF1α, which is packaged in sEV^HYP^ and predicts clinical recurrence in luminal breast cancer patients.

sEVs are recognized as pleiotropic effectors of intercellular communication in a breast cancer microenvironment (36) that affect key mechanisms of disease progression (37). Our findings highlight a unique oncogenic breadth of this pathway, especially sEVs produced by invasive breast cancer cells in hypoxia (14) to globally reprogram a normal mammary epithelium (28, 29) while also introducing systemic immunosuppression via myeloid cell release of the S100A9 alarmin (21). Mechanistically, sEV^HYP^-induced mammary gland transformation required a competent immune system in recipient animals and was abolished in mice with homozygous deletion of S100A9. Specifically for breast cancer, it is possible that S100A9-mediated immunosuppression (38) enables the liver tropism of sEV^HYP^-exposed luminal cells, while also promoting mammary gland transformation via transcriptional coactivation of cancer genes (32) and intercellular communication with transformed cells (39). Although a contribution of S100 family proteins in sEV signaling in cancer has not been previously proposed, these data fit well with other evidence that sEVs participate in tumor immune escape by affecting macrophage glycolytic metabolism (40), delivery of PD-L1 (41), and myeloid-derived suppressor cell function (42).

Against this backdrop, sEV^HYP^ signaling from invasive, but not noninvasive, breast cancer cells disrupted multiple steps of mammary gland differentiation, with expansion of MaSCs and luminal progenitors, accumulation of differentiated luminal, but not basal, cells, and deregulation of myoepithelial polarity markers. This led to unique pathologic features of mammary ductal hyperplasia and intraepithelial neoplasia, reminiscent of high-risk and difficult-to-manage breast premalignant lesions in humans (22). Although genetically stable, we found that the transcriptome of sEV^HYP^-induced lesions resembled luminal A and B breast cancer with additional similarities with the normal-like subtype (43). In line with the extraordinary plasticity of luminal progenitors (44), other features of sEV^HYP^-induced mammary gland transformation are reminiscent of luminal breast cancer, including lower expression of ER, PR, and HER2 (4), which has profound implications for patient survival (45), and dramatic acceleration of MMTV-PyMT breast tumorigenesis (35), a widely accepted model of luminal disease (46). Together, this suggests a luminal disease *acceleration* model, where sEV^HYP^ released by “stressed,” i.e., hypoxic breast cancer cells drive advanced traits of oncogenic transformation of the neighboring mammary epithelium, primed to evolve into full-blown malignancy when additional driving oncogenic signals are present (47), thus multiplying the risk of multifocal and recurrent breast cancer (23).

In this context, mammary gland lesions generated by sEV^HYP^ exhibited extensive reprogramming of multiple bioenergetics pathways. Rewiring of metabolism is an important trait of breast tumorigenesis, involving aerobic glycolysis (48), the pentose phosphate pathway (49), and mitochondrial fatty acid oxidation (50). The general activation of multiple bioenergetics pathways observed here, encompassing oxidative phosphorylation, glycolytic metabolism, cholesterol biosynthesis, and white adipose tissue browning, has not been previously associated with sEV signaling, let alone in cancer, and may be ideally positioned to fuel the expansive premalignant phenotype of atypical mammary gland hyperplasia and intraepithelial neoplasia in vivo. Consistent with this view, assembly of mitochondrial oxidative phosphorylation supercomplexes (51), deregulated lipid metabolism (52), and white adipose tissue browning (53) are all recognized drivers of breast cancer progression, immune evasion (54), and cancer-associated cachexia (55).

Mechanistically, a key mediator of both local and systemic changes introduced by sEV^HYP^ signaling was HIF1α packaged in sEV^HYP^. Pharmacologic or genetic targeting of this pathway fully reversed the ability of sEV^HYP^ to drive mammary epithelium transformation and prevented MaSC/progenitor cell expansion, associated angiogenesis, and T cell immunosuppression. Although amply studied as a master regulator of the cellular response to oxygen (30), a role of HIF1α in hypoxic sEV signaling has not, to our knowledge, been previously described, and its oncogenic activity has been mostly linked to late-stage breast cancer traits of angiogenesis (56) and metastatic competence (57). Our findings expand this paradigm, uncover a role of clathrin-mediated endocytosis (58) in selective HIF1α accumulation in hypoxic sEVs, and suggest that a new sEV^HYP^-HIF1α axis drives local and systemic mechanisms of early stages of breast tumorigenesis. One possibility is that stabilized and transcriptionally active HIF1α delivered by sEV^HYP^ creates a “pseudohypoxic” state (59) that further increases the expression of HIF1α in the mammary gland epithelium, deregulates stem/progenitor cell differentiation, and drives EMT, cell invasion, and angiogenesis. In addition, and consistent with the data presented here, there is ample evidence that HIF1α and an associated (pseudo)hypoxic environment have important roles in immune evasion (60), potentially via modulation of PD-L1 levels (61). Accordingly, sEVs generated under normoxic conditions (sEV^NORM^) did not contain HIF1α and failed to promote systemic or local changes of mammary epithelium transformation.

In addition to targeted delivery of therapeutic agents, exosomes, including sEVs, have attracted attention as readily accessible cancer biomarkers (25). Together with detection of cell-free nucleic acids (62) and circulating tumor cells (63), progress in sEV quantification in patient plasma (64) has advanced the feasibility of noninvasive “liquid biopsies” for early tumor diagnosis, staging, and assessment of residual disease, including in breast cancer (65). Consistent with this view, detection of HIF1α in plasma circulating sEVs was feasible and predicted luminal breast cancer recurrence in a small, proof-of-concept patient series, up to 103 months after diagnosis. Although these results await confirmation in larger patient cohorts, sEV-associated proteins may provide viable circulating biomarkers for various stages of breast cancer tumorigenesis (66), and the development of straightforward, point-of-service diagnostic tests for early recurrence in luminal breast cancer remains an urgent and unmet priority.

## Methods

Further information can be found in Supplemental Methods.

### Small extracellular vesicle isolation.

EO771, Brpkp110, 4T1, or AT3 cells at 70% confluence were cultured in RPMI (or DMEM/F12 for Brpkp110 cells) medium without FBS for 24 hours in normoxic or hypoxic condition (5% or 1% O_2_, respectively). pLKO-AT3 or shHIF1α-AT3 cells were cultured in RPMI medium with puromycin and without FBS for 24 hours in hypoxic condition. For small interfering RNA (siRNA) experiments, 5 × 10^5^ AT3 cells were seeded in 100 mm dishes and, after 24 hours, treated with MISSION siRNA Universal Negative Control (MilliporeSigma) or individual siRNA targeting clathrin (SASI_Mm01_00088441, clathrin 1, and SASI_Mm01_00088442, clathrin 2, MilliporeSigma). For gene silencing experiments, individual siRNA oligonucleotide sequences were transfected at 10 nM concentrations in the presence of Lipofectamine RNAiMAX in a 1:1 ratio (Invitrogen). After 24 hours the medium was replaced with RPMI without FBS, and cells were cultivated for 24 hours in hypoxia. Cells were confirmed for target protein knockdown by Western blotting, and supernatant was collected for sEV isolation. At the collection of supernatants, cells were counted, and cell death was evaluated by trypan blue staining and light microscopy (<5% dead cells). sEVs were isolated as previously described (28). Briefly, cells and debris were removed by sequential centrifugation (350*g* for 5 minutes and 1,000*g* for 10 minutes), and the supernatants were concentrated using Amicon Ultra centrifugal filter tubes (Merck Millipore). Larger vesicles were eliminated by centrifugation at 10,000*g* for 30 minutes at 4°C. Finally, extracellular vesicles were isolated by qEV size exclusion column (SEC, Izon Science), and fractions 7, 8, and 9 were collected. Isolated sEVs were stored at −80°C before analysis or protein extraction. As control, fresh RPMI medium (with or without puromycin) was processed as culture medium and stored at –80°C. The concentration and size distribution of particles were analyzed after each collection using a ZetaView NTA system (Particle Metrix). After quantification, sEVs were stored at –80°C as single-use aliquots to avoid freeze and thaw cycles, for a period not exceeding 3 months (67). Expression of sEV biomarkers CD63, CD9, and CD81 was quantified using an ExoView R100 system (NanoView Biosciences). The zeta potential of collected sEVs was measured using the ZetaView system. For experiments in vivo, aliquots (6 × 10^9^) of the sEVs collected from the various breast cancer cell types in normoxia (sEV^NORM^) or hypoxia (sEV^HYP^) conditions were injected in the abdominal mammary gland of immunocompetent C57BL/6 female mice. For coculture experiments, aliquots (10^6^) of the various sEV populations were incubated with HC11 cells (5 × 10^4^) followed by quantification of cellular responses.

### RNA isolation from sEVs.

Total RNA was isolated from 10^9^ sEVs and from the various producing cells using the Direct-zol RNA Miniprep kit (Zymo Research) according to the manufacturer’s instructions. cDNA was prepared with High-Capacity cDNA Reverse Transcription Kit with RNase Inhibitor (Thermo Fisher Scientific). Quantitative PCR reactions were performed using SYBR Green PCR Master Mix for 18S and β-actin (used as a housekeeping gene) and HIF1α (Table 1).

### Isolation of plasma sEVs.

Plasma samples were stored in 500 μL aliquots at –80°C. Plasma collected from patients with confirmed diagnosis of luminal breast cancer (7 patients with disease recurrence and 7 patients without disease recurrence) was used for subsequent analysis. For each sample, a plasma aliquot of 500 μL was processed for sEV isolation. Briefly, the plasma sample was diluted 1:1 with 0.2-μm-filtered, sterile PBS (pH 7.4) and centrifuged at 1,000 rpm for 10 minutes at 4°C to remove cells/debris and then at 10,000 rpm for 30 minutes at 4°C to remove large EVs. sEVs were isolated by qEV size exclusion column (SEC, Izon Science) with collection of fractions 7, 8, and 9. Isolated sEVs were stored at −80°C before analysis or protein extraction. The concentration and size distribution of particles were analyzed after each collection using the ZetaView NTA system (Particle Metrix). To increase the purity of the patient-derived sEV preparation, an EpCAM Exo-Flow Capture Kit (System Biosciences) was used following the manufacturer’s instructions. Briefly, magnetic beads coated with an antibody against EpCAM (40 μL) were incubated with 500 μL aliquots of isolated plasma-derived sEVs on a rotating rack for 12 hours at 4°C to enrich the population of EpCAM^+^ sEVs. To confirm binding, sEVs were incubated with Exo-FITC exosome stain for 2 hours at 0°C and analyzed by flow cytometry. Finally, beads-sEVs were lysed with 100 μL of RIPA buffer and incubated on a rotating rack for protein isolation for 12 hours at 4°C.

### Bioinformatics.

RNA-Seq data were aligned using the STAR (68) algorithm against the mm10 version of mouse genome, and RSEM v1.2.12 software (66) was used to estimate read counts and fragments per kilobase per million total reads (FPKM) values using gene information from the Ensembl transcriptome version GRCm38.89. Raw counts were used to estimate significance of difference in expression between 2 experimental groups using DESeq2 (69), and normalized DESeq2 counts were used to generate expression heatmaps. Gene set enrichment analysis was done with Ingenuity Pathway Analysis (IPA) software (QIAGEN, www.qiagen.com/ingenuity) using “canonical pathways,” “diseases & functions,” and “upstream regulators” options. Significant results at *P* less than 0.05 with a predicted activation state (|*z*| > 1) were considered. RNA-Seq data were submitted to the NCBI’s Gene Expression Omnibus (GEO) database under accession number GSE225986.

Whole genome sequencing was performed for control (*n* = 2) and sEV^HYP^ (*n* = 3) conditions. The data were aligned against the mm10 version of genome using the Burrows-Wheeler Aligner (BWA) algorithm (70), and mutation calls were performed for control and sEV^HYP^ samples versus first and second control replicate separately using VarScan v2.3.9 (71) with default parameters. Only significant (Fisher’s exact test *P* < 0.05) mutations supported by at least 5 reads with no more than 2 alternative reads in the control samples were considered. Effect of called mutations on protein level was annotated using SnpEff v2.3 (72). Average number of mutations between use of control 1 and use of control 2 as a reference was reported. TCGA breast cancer (BRCA) expression data set with tumors’ subtypes was downloaded from cBioPortal (73). The expression levels of genes from the PAM50 panel (74) were used to calculate similarity of sample profiles using Spearman’s correlation.

A potential relationship between luminal cells treated with sEV^HYP^ or sEV^NORM^ and individual breast cancer subtypes was tested versus the TCGA and CCLE databases. TCGA breast cancer (BRCA) expression data set with tumors’ subtypes was downloaded from cBioPortal (73). Gene expression levels for cell lines in the CCLE database were downloaded from the DepMap portal (https://depmap.org/portal/download/; data set DepMap Public 20Q4 v2). The expression levels of genes differentially expressed between sEV^HYP^ and sEV^NORM^ conditions were *z* score–transformed for luminal cells, TCGA samples, and CCLE samples independently. For TCGA patient data, all sample profiles were projected on principal components found from principal component analysis (PCA) of sEV^HYP^ versus sEV^NORM^ samples. For the CCLE data set, PCA was carried out using samples from luminal and CCLE data sets together, and principal components 2 and 3 were selected for sample classification, as the first principal component reflected differences between the 2 experiments.

### Statistics.

Statistical analysis was performed using GraphPad Prism 9 software. All biological experiments were performed at least 3 times (all single experiments had a technical duplicate). To compare means for 2 groups, a Mann-Whitney 2-tailed *t* test with Tukey’s post-test was performed. For comparison of 3 or more groups, a 1-way ANOVA test was used. To determine how a response was affected by 2 factors, a 2-way ANOVA test was used.

### Study approval.

Albino-C57BL/6 [B6(Cg)-Tyrc-2J/J], BALB/cJ, and MMTV-PyMT (stock 002374) mice (75) were obtained from The Jackson Laboratory. NOD SCID γ (NSG; NOD.Cg-Prkdcscid IL2rγtm1Wjl/SzJ) mice were bred in the pathogen-free animal facility at The Wistar Institute. All experiments were approved by The Wistar Institute Institutional Animal Care and Use Committee. Aliquots of patient plasma were collected at diagnosis at The Research Institute of Fox Chase Cancer Center (Philadelphia, Pennsylvania, USA) on behalf of the Biosample Repository Facility and transferred to The Wistar Institute under approved material transfer agreement 22011337.

## Author contributions

IB and DCA conceived the project. IB performed experiments of sEV characterization, signaling, and modulation of luminal mammary epithelium reprogramming. MP characterized sEV regulation of mammary gland stem and progenitor cell differentiation and EMT induction. AM evaluated sEV-induced metabolic reprogramming of the mammary epithelium. PV evaluated mammary gland histology and immunohistochemistry. YN and CL contributed experiments with S100A9-knockout mice, and AVK analyzed bioinformatics data. JCG generated the shHIF1α stable cell lines. IB, MP, AVK, PV, and DCA analyzed data, and IB and DCA wrote the manuscript.

## Supplementary Material

Supplemental data

Supplemental table 1

Supplemental table 2

Supplemental table 3

## Figures and Tables

**Table 1:**
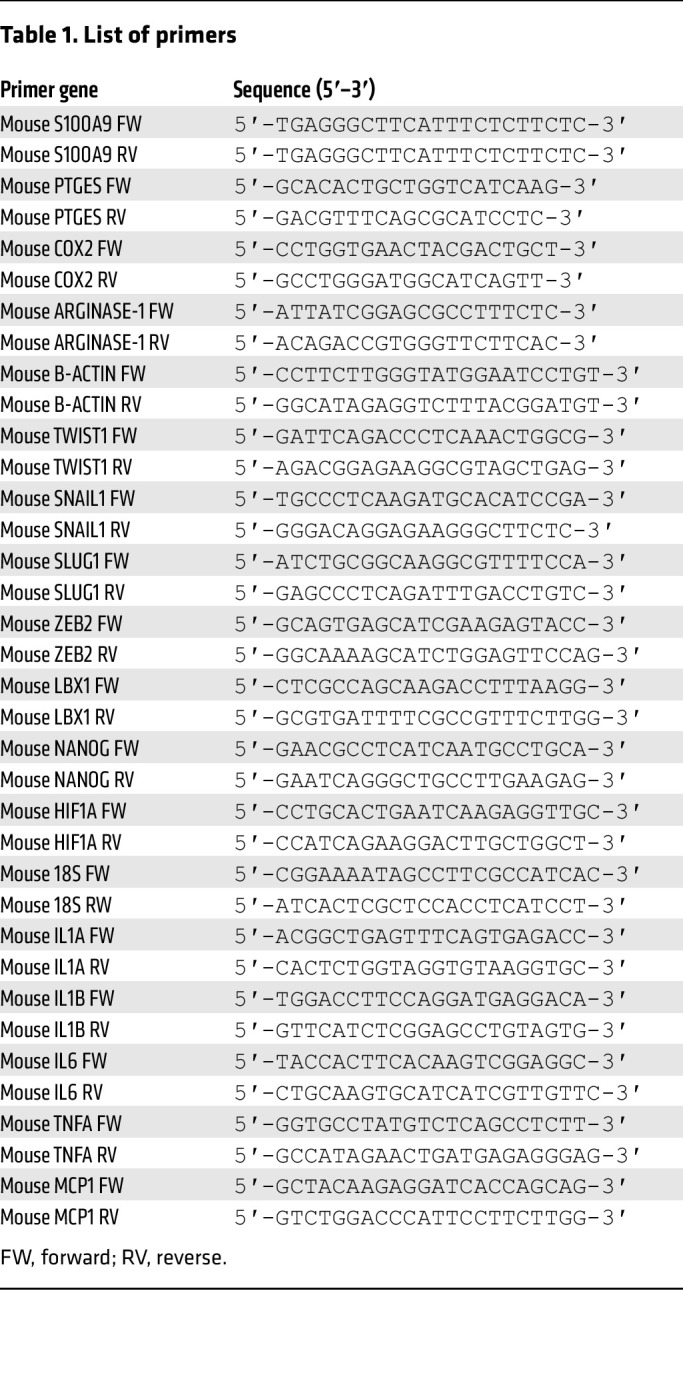
List of primers

## Version 1 03/09/2023

In-Press Preview

## Version 2 04/17/2023

Electronic publication

## References

[1] DeSantis CE (2019). Breast cancer statistics, 2019. CA Cancer J Clin.

[2] Zardavas D (2015). Clinical management of breast cancer heterogeneity. Nat Rev Clin Oncol.

[3] Polyak K (2007). Breast cancer: origins and evolution. J Clin Invest.

[4] Harbeck N (2019). Breast cancer. Nat Rev Dis Primers.

[5] Slepicka PF (2021). The molecular basis of mammary gland development and epithelial differentiation. Semin Cell Dev Biol.

[6] Keller PJ (2012). Defining the cellular precursors to human breast cancer. Proc Natl Acad Sci U S A.

[7] Visvader JE, Stingl J (2014). Mammary stem cells and the differentiation hierarchy: current status and perspectives. Genes Dev.

[8] Hanahan D, Coussens LM (2012). Accessories to the crime: functions of cells recruited to the tumor microenvironment. Cancer Cell.

[9] Quail DF, Joyce JA (2013). Microenvironmental regulation of tumor progression and metastasis. Nat Med.

[10] Shibue T, Weinberg RA (2017). EMT, CSCs, and drug resistance: the mechanistic link and clinical implications. Nat Rev Clin Oncol.

[11] Lehuede C (2016). Metabolic plasticity as a determinant of tumor growth and metastasis. Cancer Res.

[12] Kim C (2018). Chemoresistance evolution in triple-negative breast cancer delineated by single-cell sequencing. Cell.

[13] Semenza GL (2013). HIF-1 mediates metabolic responses to intratumoral hypoxia and oncogenic mutations. J Clin Invest.

[14] Zhang Y (2021). Hypoxia in breast cancer-scientific translation to therapeutic and diagnostic clinical applications. Front Oncol.

[15] Bos R (2001). Levels of hypoxia-inducible factor-1 alpha during breast carcinogenesis. J Natl Cancer Inst.

[16] Baldominos P (2022). Quiescent cancer cells resist T cell attack by forming an immunosuppressive niche. Cell.

[17] Binnewies M (2018). Understanding the tumor immune microenvironment (TIME) for effective therapy. Nat Med.

[18] Tcyganov E (2018). Plasticity of myeloid-derived suppressor cells in cancer. Curr Opin Immunol.

[19] Ali HR (2016). Patterns of immune infiltration in breast cancer and their clinical implications: a gene-expression-based retrospective study. PLoS Med.

[20] DeNardo DG (2011). Leukocyte complexity predicts breast cancer survival and functionally regulates response to chemotherapy. Cancer Discov.

[21] Bresnick AR (2015). S100 proteins in cancer. Nat Rev Cancer.

[22] Morrow M (2015). Current management of lesions associated with an increased risk of breast cancer. Nat Rev Clin Oncol.

[23] Curtius K (2018). An evolutionary perspective on field cancerization. Nat Rev Cancer.

[24] Jaiswal R, Sedger LM (2019). Intercellular vesicular transfer by exosomes, microparticles and oncosomes — implications for cancer biology and treatments. Front Oncol.

[25] Kalluri R, LeBleu VS (2020). The biology, function, and biomedical applications of exosomes. Science.

[26] Antonyak MA (2011). Cancer cell-derived microvesicles induce transformation by transferring tissue transglutaminase and fibronectin to recipient cells. Proc Natl Acad Sci U S A.

[27] Sung BH (2021). Extracellular vesicles: critical players during cell migration. Dev Cell.

[28] Bertolini I (2020). Small extracellular vesicle regulation of mitochondrial dynamics reprograms a hypoxic tumor microenvironment. Dev Cell.

[29] Bertolini I (2022). NFkappaB activation by hypoxic small extracellular vesicles drives oncogenic reprogramming in a breast cancer microenvironment. Oncogene.

[30] de Heer EC (2020). HIFs, angiogenesis, and metabolism: elusive enemies in breast cancer. J Clin Invest.

[31] Thery C (2018). Minimal information for studies of extracellular vesicles 2018 (MISEV2018): a position statement of the International Society for Extracellular Vesicles and update of the MISEV2014 guidelines. J Extracell Vesicles.

[32] Song R, Struhl K (2021). S100A8/S100A9 cytokine acts as a transcriptional coactivator during breast cellular transformation. Sci Adv.

[33] Zhao X (2012). TNF signaling drives myeloid-derived suppressor cell accumulation. J Clin Invest.

[34] Ham B (2015). TNF receptor-2 facilitates an immunosuppressive microenvironment in the liver to promote the colonization and growth of hepatic metastases. Cancer Res.

[35] Attalla S (2021). Insights from transgenic mouse models of PyMT-induced breast cancer: recapitulating human breast cancer progression in vivo. Oncogene.

[36] Lucien F, Leong HS (2019). The role of extracellular vesicles in cancer microenvironment and metastasis: myths and challenges. Biochem Soc Trans.

[37] Sansone P (2017). Packaging and transfer of mitochondrial DNA via exosomes regulate escape from dormancy in hormonal therapy-resistant breast cancer. Proc Natl Acad Sci U S A.

[38] Li J (2022). S100A9-CXCL12 activation in BRCA1-mutant breast cancer promotes an immunosuppressive microenvironment associated with resistance to immunotherapy. Nat Commun.

[39] Jo SH (2021). S100A8/A9 mediate the reprograming of normal mammary epithelial cells induced by dynamic cell-cell interactions with adjacent breast cancer cells. Sci Rep.

[40] Morrissey SM (2021). Tumor-derived exosomes drive immunosuppressive macrophages in a pre-metastatic niche through glycolytic dominant metabolic reprogramming. Cell Metab.

[41] Chen G (2018). Exosomal PD-L1 contributes to immunosuppression and is associated with anti-PD-1 response. Nature.

[42] Chalmin F (2010). Membrane-associated Hsp72 from tumor-derived exosomes mediates STAT3-dependent immunosuppressive function of mouse and human myeloid-derived suppressor cells. J Clin Invest.

[43] Cancer Genome Atlas Network (2012). Comprehensive molecular portraits of human breast tumours. Nature.

[44] Lim E (2009). Aberrant luminal progenitors as the candidate target population for basal tumor development in BRCA1 mutation carriers. Nat Med.

[45] Lindstrom LS (2018). Intratumor heterogeneity of the estrogen receptor and the long-term risk of fatal breast cancer. J Natl Cancer Inst.

[46] Herschkowitz JI (2007). Identification of conserved gene expression features between murine mammary carcinoma models and human breast tumors. Genome Biol.

[47] D’Cruz CM (2001). c-MYC induces mammary tumorigenesis by means of a preferred pathway involving spontaneous Kras2 mutations. Nat Med.

[48] Chen F (2019). Extracellular vesicle-packaged HIF-1α-stabilizing lncRNA from tumour-associated macrophages regulates aerobic glycolysis of breast cancer cells. Nat Cell Biol.

[49] Kim NH (2017). Snail reprograms glucose metabolism by repressing phosphofructokinase PFKP allowing cancer cell survival under metabolic stress. Nat Commun.

[50] Almanza A (2022). Regulated IRE1α-dependent decay (RIDD)-mediated reprograming of lipid metabolism in cancer. Nat Commun.

[51] Ikeda K (2019). Mitochondrial supercomplex assembly promotes breast and endometrial tumorigenesis by metabolic alterations and enhanced hypoxia tolerance. Nat Commun.

[52] Nelson ER (2013). 27-Hydroxycholesterol links hypercholesterolemia and breast cancer pathophysiology. Science.

[53] Qiang L (2012). Brown remodeling of white adipose tissue by SirT1-dependent deacetylation of Pparγ. Cell.

[54] Huang B (2020). Cholesterol metabolism in cancer: mechanisms and therapeutic opportunities. Nat Metab.

[55] Petruzzelli M (2014). A switch from white to brown fat increases energy expenditure in cancer-associated cachexia. Cell Metab.

[56] Casillas AL (2021). Direct phosphorylation and stabilization of HIF-1α by PIM1 kinase drives angiogenesis in solid tumors. Oncogene.

[57] Yang MH (2008). Direct regulation of TWIST by HIF-1alpha promotes metastasis. Nat Cell Biol.

[58] Ginini L (2022). Insight into extracellular vesicle-cell communication: from cell recognition to intracellular fate. Cells.

[59] Leontieva OV, Blagosklonny MV (2014). M(o)TOR of pseudo-hypoxic state in aging: rapamycin to the rescue. Cell Cycle.

[60] Hatfield SM (2015). Immunological mechanisms of the antitumor effects of supplemental oxygenation. Sci Transl Med.

[61] Bailey CM (2022). Targeting HIF-1α abrogates PD-L1-mediated immune evasion in tumor microenvironment but promotes tolerance in normal tissues. J Clin Invest.

[62] Wan JCM (2017). Liquid biopsies come of age: towards implementation of circulating tumour DNA. Nat Rev Cancer.

[63] Cristofanilli M (2004). Circulating tumor cells, disease progression, and survival in metastatic breast cancer. N Engl J Med.

[64] Cheng L, Hill AF (2022). Therapeutically harnessing extracellular vesicles. Nat Rev Drug Discov.

[65] Dong M (2022). Extracellular vesicles: the landscape in the progression, diagnosis, and treatment of triple-negative breast cancer. Front Cell Dev Biol.

[66] Li D (2021). Protein biomarkers in breast cancer-derived extracellular vesicles for use in liquid biopsies. Am J Physiol Cell Physiol.

[67] Gelibter S (2022). The impact of storage on extracellular vesicles: a systematic study. J Extracell Vesicles.

[68] Dobin A (2013). STAR: ultrafast universal RNA-seq aligner. Bioinformatics.

[69] Love MI (2014). Moderated estimation of fold change and dispersion for RNA-seq data with DESeq2. Genome Biol.

[70] Li H, Durbin R (2010). Fast and accurate long-read alignment with Burrows-Wheeler transform. Bioinformatics.

[71] Koboldt DC (2012). VarScan 2: somatic mutation and copy number alteration discovery in cancer by exome sequencing. Genome Res.

[72] Cingolani P (2012). A program for annotating and predicting the effects of single nucleotide polymorphisms, SnpEff: SNPs in the genome of Drosophila melanogaster strain w1118; iso-2; iso-3. Fly (Austin).

[73] Cerami E (2012). The cBio cancer genomics portal: an open platform for exploring multidimensional cancer genomics data. Cancer Discov.

[74] Bastien RR (2012). PAM50 breast cancer subtyping by RT-qPCR and concordance with standard clinical molecular markers. BMC Med Genomics.

[75] Guy CT (1992). Induction of mammary tumors by expression of polyomavirus middle T oncogene: a transgenic mouse model for metastatic disease. Mol Cell Biol.

