# Supplementary material for: Intercellular hif1α reprograms mammary progenitors and myeloid immune evasion to drive high-risk breast lesions
Source: J Clin Invest. 2023 Apr 17;133(8):e164348. doi: 10.1172/JCI164348 (PMC10104898; doi:10.1172/JCI164348)
Supplement: Supplemental data [file jci-133-164348-s140.pdf]

Rev Ms 164348-JCI-RG-2

INTERCELLULAR HIF1α REPROGAMS MAMMARY PROGENITORS AND MYELOID IMMUNE EVASION TO DRIVE HIGH-RISK BREAST LESIONS

Irene Bertolini, Michela Perego, Yulia Nefedova, Cindy Lin, Andrew Milcarek, Peter Vogel, Jagadish C. Ghosh, Andrew V. Kossenkov and Dario C. Altieri

SUPPLEMENTAL INFORMATION

SUPPLEMENTAL METHODS

Antibodies and reagents. Vybrant™ DiD Cell-Labeling Solution, Aqua live/dead and Fc block were purchased from ThermoFisher. Growth Factor-Reduced Matrigel was obtained from BD Biosciences (BD No. 354230). Gentle Collagenase/Hyaluronidase, Dispase (1U/ml) and DNase I solution were purchased from StemCell Technologies. Antibodies to HIF1a (# 36169), hydroxy-HIF1a (# 3434), PHD-2 (#4835), Ser473-phosphorylated AKT (# 9271), AKT (# 9272), Thr202/Tyr204-phosphorylated ERK1/2 (# 4370), ERK1/2 (# 4695), MMP9 (# 13667), Calnexin (# 2679), Flotillin-1 (# 18634), GM130 (# 12480), SNAIL (# 3879), E-cadherin (# 3195), N-cadherin (# 13116), β-actin (# 5125), CD31 (# 77699) and Ki67 (# 12202) were obtained from Cell Signaling. Antibodies to Tsg101 (# 14497-1-AP), Cytokeratin 8 (CK8, # 17514-1-AP), Cytokeratin 14 (CK14, # 60320-1-Ig), UCP1 (# 23673-1-AP), SMMHC (# 21404-1-AP), Clathrin (# 10852-1-AP), HIF1α (# 20960-1-AP) and p63 (# 12143-1-AP) were from ProteinTech. Antibodies to CD63 (# ab59479) and CD9 (# ab223052) were purchased from Abcam. Antibodies to Epcam (# 118213), CD24 (# 138503) and CD49f (# 313621), CD11b (# 101236), CD8 (# 100753) were obtained from Biolegend. Antibodies to CD45 (# 560510), Ly6G (# 551460), Ly6C (# 560593), CD3 (#553063) were obtained from BD. Antibodies to F4/80 (#12-4801-82), CD11c (# 17-0114-82), CD4 (#17-0041-83), B220 (# 60-0452-Uo25) were obtained from eBioscience. The small molecule AKT inhibitor MK2206 (1 μM) and MEK/ERK inhibitor PD98059 (10 μM) were purchased from Selleck Chemicals and HIF1α inhibitor PX-478 was purchased from MedChemExpress.

Cell culture. The AT3 mouse mammary adenocarcinoma cell line was obtained from MilliporeSigma. Non-invasive and non-metastatic EO771 mouse mammary tumor cells, 4T1 metastatic mammary carcinoma cells and normal mammary epithelial HC11 cells were obtained

from the American Type Culture Collection (ATCC, Manassas, VA). Brpkp110 mouse mammary carcinoma cells were provided by Dr. Schug (The Wistar Institute). AT3, 4T1, EO771 and HC11 cells were maintained in culture in RPMI 1640 medium supplemented with 10% fetal bovine serum (FBS) plus 1% streptomycin and penicillin. Brpkp110 cells were maintained in DMEM/F12 medium supplemented with 10% fetal bovine serum (FBS) plus 1% streptomycin and penicillin. Conditioned media used for cell migration assays were prepared from exponentially growing cultures of NIH3T3 cells (ADCC) in Dulbecco's modified Eagle's medium (DMEM) supplemented with 4.5 g/l D-glucose, sodium pyruvate, 10 mM HEPES, and 10% FBS for 48 h. All cell lines were maintained at 37°C.

Generation of shHIF1α stable cell line. The lentiviral plasmid MISSION® pLKO.1 or HIF1a shRNA were purchased by MilliporeSigma. Mission pLKO.1-puro Non-Mammalian shRNA Control Plasmid DNA # SHC002; HIF1α shRNA-0 #TRCN0000232222; HIF1α shRNA-1 #TRCN0000232223; and HIF1α shRNA-2 # TRCN0000232220. The plasmids were co-transfected in 293T cells using lentiviral packaging plasmids. Lentiviral supernatant was harvested 48 h after transfection and stored at -80°C. AT3 cells stably expressing MISSION® pLKO.1 or HIF1a shRNA were generated by infection with lentiviral particles, selected throughout a 2-week culture in the presence of puromycin (2 μg/ml), and individual clones were established and further characterized for HIF1a silencing, by Western blotting.

Cellular respiration. Oxygen consumption rates (OCR) or extracellular acidification rates (ECAR) were quantified in HC11 cells treated with AT3 cell-derived sEV for 24 h using an Agilent Seahorse XFe96 analyzer (Agilent Technologies, Wilmington, DE). Metabolic rates were monitored under basal conditions and after addition of oligomycin (1 μM), FCCP (1 μM) and antimycin (0.5 μM), all dissolved in XF base media. After sequential drug addition,

OCR/ECAR rates were measured using three cycles of mixing (150 sec), waiting (120 sec) and measuring (210 sec). This cycle was repeated following each injection.

Cell migration. Cell migration experiments were performed essentially as described (1) using PET Transwell chambers (Corning). Isolated luminal cells established as described above were seeded on Transwell filters ($5x10^4$ cells/well) in medium containing 0.1% bovine serum albumin (BSA). Aliquots of conditioned medium from NIH3T3 fibroblasts were placed in the lower chamber as a chemoattractant. Cells were allowed to invade or migrate for 24 h, non-invading cells were removed whereas invaded cells were fixed in methanol. Membranes were mounted in medium containing DAPI (Vector Laboratories) and analyzed by fluorescence microscopy. Ten fields at 10X magnification were collected and the number of invading or migrating cells was quantified using ImageJ.

3D colony formation. 3D culture of isolated luminal cells was performed as described (1). Each well of a µ-Slide 8 Well chamber (Ibidi) was coated with 40 µl of Growth Factor-Reduced Matrigel (BD Biosciences). Luminal cells ($5x10^3$/well) were suspended in Assay Medium (DMEM/F12, 2% horse serum, 0.5 µg/ml hydrocortisone, 100 ng/ml cholera toxin, 10 µg/ml insulin and pen/strep) plus 2% Matrigel and 5 ng/ml EGF and seeded on coated Matrigel. Cultures were supplemented with assay medium containing 2% Matrigel and 5 ng/ml EGF every 4 d. Brightfield images were collected at d. 7 or d. 14 using a TE300 inverted microscope (Nikon) equipped with an incubator set at 37°C with 5% CO_2 and 95% relative humidity with a 10X objective.

Luminal cell ex vivo culture. Sorted mammary gland luminal cells were washed, counted and seeded in DMEM-F12 medium enriched with 5% horse serum (HS), 20 ng/ml epidermal growth factor (EGF), 0.5 mg/ml hydrocortisone, 100 ng/ml cholera toxin, 10 µg/ml insulin and 1%

streptomycin and penicillin. After 24 h, the medium was changed and replaced with DMEM F12 without serum to avoid expansion of stromal cells. The culture medium was replaced every 2 d and the number of passages in culture was monitored. Cell purity (>85%) was assessed from the expression of luminal cells markers $CD45^-$, $EpCAM^+$ and $CD49f^+$) by flow cytometry.

Luminal cell in vivo experiment. Sorted mammary gland luminal cells were stained with DiD-Vybrant dye (Thermo Fisher) for 30min at 37°C and then washed twice in DPBS. One hundred thousand cells resuspended in 100 µl of PBS: Matrigel (1:1) were injected in the abdominal gland of NOD-SCID $IL2Rg^{null}$ or C57BL/6 mice (pre conditioned for 5 weeks with $6x10^9$ sEV^{CTRL} or sEV^{HYP}). Cells were tracked using an IVIS® SpectrumCT In Vivo Imaging System at the time of injection and after 5 d. Mice were sacrificed 8 weeks after luminal cells injection and organs were harvested.

PMN magnetic isolation. PMN were isolated from tumor free (naïve) mice spleen. Spleens were harvested, mechanically dissociated and after red blood cells lysis with ACK buffer (Qality Biological) cells were stained with mouse microbeads anti-Ly6G antibody 15 min at 4°C (Miltenyi Biotech, Bergisch Gladbach, Germany). $Ly6G^+$ cells were purified by magnetic separation (Miltenyi Biotec) according to the manufacturer's instructions.

Suppression assay. $CD8^+$ cells were isolated from tumor-free mouse spleens. Samples were harvested, mechanically dissociated and after RBC lysis with ACK buffer, cells were stained with FC block for 5 min at 4°C. After FC incubation, a combination of Aqua live dead dye (1:300) and rat-anti mouse CD8 at 0.5 µl/$1x10^6$ cells ratio was added and incubated for 15 min at 4°C. After washing, cells were filtered through a CellTriCS ™ filter (Sysmec, Goerlitz, Germany) and $CD8^+$ cells were purified after exclusion of dead cells and doublets by sorting using a MoFlo Astrios Cell sorter (Beckman Coulter). PMN were isolated by magnetic

separation as described above from tumor-free mouse spleens, and incubated with 10^7 sEVs/10^5 PMN in compete medium (RPMI+10%FBS) supplemented with 10 ng/ml GM-CSF (Peprotech) for 4 h at 37°C. After incubation, PMN were washed and plated for suppression assay at the indicated ratio with splenocytes activated with CD3/CD28 Dynabeads™. Splenocytes were obtained by mechanical dissociation of tumor-free mouse spleens and labeled with CellTrace™ Violet (ThermoFisher). Forty-eight h after activation, cells were harvested, stained with antibodies to CD4, CD8 and Ly6G and acquired on a BD FACSCelesta™ flow cytometer Percentages of proliferating cells were calculated using the FlowJo software.

qRT-PCR. PMN sorted from mammary gland were stored in mirVANA™ lysis buffer at -20°C. RNA was then extracted according to the mirVANA™ miRNA Isolation kit (ThermoFisher Scientific) protocol. Luminal and basal cells isolated from sEV-injected mammary glands as described above were stored at -80°C in Quick-RNA™ Microprep Kit lysis buffer (Zymo Research, Irvine, CA). Alternatively, luminal cells were maintained in culture for 1 week and then RNA was extracted according to the kit protocol. cDNA was prepared with High-Capacity cDNA Reverse Transcription Kit with RNase Inhibitor (ThermoFisher Scientific). qPCR reactions were performed using SYBR™ Green PCR Master Mix for selected genes of interest using an ABI Quant Studio 5 Real-Time PCR Systems (ThermoFisher Scientific).

ELISA of S100A9. Mouse peripheral blood was collected from intracardiac puncture. Blood was centrifugated for 15’ at 2000 g in refrigerated centrifuge and stored at -20C°. S100A8/A9 was measured by Mouse S100A8/S100A9 Heterodimer DuoSet ELISA (R&D System, Minneapolis, MN, USA) according to manufacturer instruction.

Flow cytometry and sorting. Mammary glands were harvested from the various animal groups and digested to obtain single cell suspensions. The protocol was adapted from the StemCell

protocol available online to isolate mouse mammary epithelial cells, Briefly, mammary glands were first mechanically dissociated and then enzymatically dissociated through incubation with Gentle Collagenase/Hyaluronidase for 3 h at 37°C. After processing, all samples were filtered through a 70 μM filter (WWR) and red blood cells lysis was performed in the presence of ACK buffer for 2 min at 22°C. For flow cytometry analysis, cells were incubated with FC block (BD) reagent for 5 min before addition of antibody mixture (Aqua Live/Dead 1:300, CD45 1:100, EpCAM 1:100, CD24 1:100 and CD49f 1:200) for 15 min at 4°C. The individual mammary gland subsets were identified using the following markers as described in published protocols (2): Mammary stem cells (MaSC): $CD45^{-}/EpCAM^{-}/CD49f^{HIGH}/CD24^{LOW}$; Luminal 1 (L1) progenitor cells: $CD45^{-}/EpCAM^{+}/CD49f^{+}/CD24^{HIGH}$; Luminal 2 (L2) progenitor cells: $CD45^{-}/EpCAM^{+}/CD49f^{LOW}$; Luminal cells: $CD45^{-}/EpCAM^{+}/CD49f^{+}$; Basal cells: $CD45^{-}/EpCAM^{-}/CD49f^{+}$. For sorting, a single cell suspension was incubated with mouse FC block reagent and then Aqua Live/Dead dye (1:300) for 5 min at 4°C. After addition of antibodies to EpCAM, CD49f, and CD45 (0.5 μl/16 cells), the different populations were isolated by fluorescence-activated cell sorting with gating on live single cells using a MoFLoAstrios flow cytometer.

IVIS SpectrumCT analysis. Aliquots of sEV ($6x10^{9}$) were injected s.c. in the right abdominal mammary gland of female C57BL/6 mice followed by i.v. administration (100 μl) of IVISense Vascular 750 Fluorescent Probe after 1, 2, 4 and 6 weeks. Changes in angiogenesis under the various conditions were quantified by live imaging using a IVIS® SpectrumCT and 3D images were generated by integrating low-dose μCT scan. During scans, mice were kept under isoflurane anesthesia. Data obtained by IVIS analysis were subsequently analyzed with the “living image” software (PerkinElmer).

Whole genome sequencing. Genomic DNA from sorted luminal cells as above was extracted using QIAamp DNA mini kit (Qiagen) according to the manufacturer's instructions. gDNA quality was assessed on a TapeStation system using gDNA ScreenTape. Whole Genome libraries were made using an Illumina DNA Prep kit in combination with DNA/RNA UD indexes. Input gDNA for library preparation was 250 ng with a final PCR amplification of 5 cycles. Library size was assessed using a 2100 Bioanalzyer and High-Sensitivity DNA assay. Concentration was determined using Qubit Fluorometer 2.0. NGS was done on a NovaSeq 6000 instrument (Illumina, San Diego, CA) using a single lane of the S4 flow cell. The sequencing was a paired-end 2x150 bp run.

RNA-Seq. Whole mouse mammary glands harvested from the various animal groups 6 weeks after sEV injection were homogenized using a hand-held Tissue Ruptor II homogenizer (Qiagen, Germantown, MD) while submerged and simultaneously lysed in TRI Reagent (Sigma, St. Louis, MO). Sorted luminal cells were also lysed in TRI reagent prior to RNA extraction. RNA from lysed mammary gland tissue or sorted luminal cells was extracted using the Direct-zol RNA Miniprep kit (Zymo Research, Irvine, CA) according to the manufacturer's instructions. After on-column DNaseI treatment, RNA integrity was assessed using the TapeStation RNA ScreenTape (Agilent, Santa Clara, CA) and concentration was determined using the Qubit 2.0 Fluorometer (ThermoFisher, Waltham, MA). Libraries for whole transcriptome RNA-Seq were prepared using the Sense mRNA-Seq Library Prep Kit V2 (Lexogen, Vienna, Austria) according to the manufacturer's instructions starting with an input of 300 ng of RNA and 13 cycles of final PCR amplification. Library size was assessed using a 2100 Bioanalzyer and High-Sensitivity DNA assay (Agilent, Santa Clara, CA). The library concentration was determined using Qubit Fluorometer 2.0 and Next Generation Sequencing (NGS) was carried out on a NextSeq500

platform (Illumina, San Diego, CA). The sequencing run was a paired-end 2x75 bp High output run.

Mouse strains and in vivo experiments. Six-week-old female mice were used for all experiments. Mice were kept on alfa-alfa free diet to avoid fluorescent background during in vivo imaging. For in vivo experiments, sEV ($6x10^9$) isolated from AT3, EO771 and Brpkp110 cells were injected subcutaneously in the right abdominal mammary gland of female albino-C57BL/6 mice. sEV ($6x10^9$) isolated from 4T1 were injected in the right abdominal gland of BALB/cJ mice. Animals were euthanized after 3 (n=30), 6 (n=30) or 18 (n=30) weeks after sEV injection mouse and organs were harvested for additional experiments. The estrus cycle of the mice was checked at the end of each experiment (Supplemental Table 1). Luminal cells sorted from the mammary gland of C57BL/6 mice 6 weeks after sEV injection were expanded ex-vivo, labeled with Vybrant™ DiD Cell-Labeling Solution to allow in vivo tracking and re-injected s.c. into the right abdominal mammary gland of NSG mice at $5x10^5$ cells/mouse (cells:Matrigel=1:1). Mice were monitored by live imaging weekly using an IVIS imaging system Living Image Software 4.7.4 (PerkinElmer), and the photon count in areas of interest was quantified after background subtraction. Mice were euthanized 8 weeks after sEV injection mice, and harvested organs were imaged as described above.

PX-478 in vivo treatment. A melphalan-derived small molecule HIF1α inhibitor PX-478 was diluted at a final concentration of 20 mg/ml in 10% DMSO and 90% 20%-SBE-β-CD in saline. PX-478 was further diluted 1:2 in saline at a final concentration of 10 mg/ml before administration, in vivo. Mice were treated i.p. with PX-478 (50 mg/kg) on d. 1 and d. 3 after sEV injection (sEV^{CTRL} or sEV^{HYP}) and monitored for 3 weeks. Control mice were injected with

vehicle. At the end of the experiment, mammary glands from the four animal groups (n = 20) were harvested and analyzed by immunohistochemistry (IHC).

Tissue immunofluorescence. Mammary glands and livers harvested from animal groups injected with the various sEV were fixed for 12 h at 4°C in 4% formaldehyde solution (pH 6.9) and then dehydrated on 30% sucrose for 24 h at 4°C. Tissues samples were embedded in O.C.T. compound and stored at -80°C. Eight-µm slides were cut and processed for staining. Mammary glands tissue slides were quenched with 0.3 M glycine for 10 min at 22°C, permeabilized with 0.5% Triton X-100 for 10 min at 22°C and blocked with 5% BSA in PBS- 0.1% Triton X-100. Primary antibodies diluted in 5% BSA (CK8 1:200; CK14 1:200) were applied for 12 h at 4°C. Secondary antibodies of appropriate specificity (goat anti-mouse AF488 and goat anti-rabbit AF568) were diluted in PBS and incubated for 1 h at 22°C. Nuclei were stained with Hoechst (1:1000) for 5 min at 22°C. Slides were acquired using a Nikon Ti Automated Inverted Microscope at 40X magnification.

IHC. Tissues harvested from the various animal groups were fixed in 10% formalin for 12 h at 22°C, rinsed in 70% EtOH, and paraffin embedded. Samples were processed for deparaffinization and rehydration in Xylene for 6 min, two changes in Xylene substitute for 5 min each, and 2 changes (2 min each) of 100% EtOH, 95% EtOH and distilled water. Antigen retrieval was carried out in Dako/EDTA solution (pH 9) in pressure cooker. Slides were cooled on counter for 30 min in retrieval reagent and washed in distilled water for 2 min. After quenching for 10 min in 3% H_2O_2, slides were washed in distilled water for 2 min and PBST (PBS-Tween) 3 times for 5 min, permeabilized with Triton X-100 for 15 min, and blocked with Horse Serum (2.5%) for 1 h. Primary antibodies to HIF1α (1:1600), Ki67 (1:200), p63 (1:300), SMMHC (1:750), CD31 (1:100), or UCP1 (1:600) were incubated for 12 h at 4°C. Secondary

antibodies of appropriate specificity were applied for 30 min at 22°C, washed and slides were counterstained with hematoxylin before light microscopy examination.

SUPPLEMENTAL REFERENCES

1. Bertolini, I., Ghosh, J. C., Kossenkov, A. V., Mulugu, S., Krishn, S. R., Vaira, V., Qin, J., Plow, E. F., Languino, L. R., and Altieri, D. C. (2020) Small Extracellular Vesicle Regulation of Mitochondrial Dynamics Reprograms a Hypoxic Tumor Microenvironment. *Dev Cell* **55**, 163-177 e166

2. Ghebeh, H., Sleiman, G. M., Manogaran, P. S., Al-Mazrou, A., Barhoush, E., Al-Mohanna, F. H., Tulbah, A., Al-Faqeeh, K., and Adra, C. N. (2013) Profiling of normal and malignant breast tissue show CD44high/CD24low phenotype as a predominant stem/progenitor marker when used in combination with Ep-CAM/CD49f markers. *BMC Cancer* **13**, 289

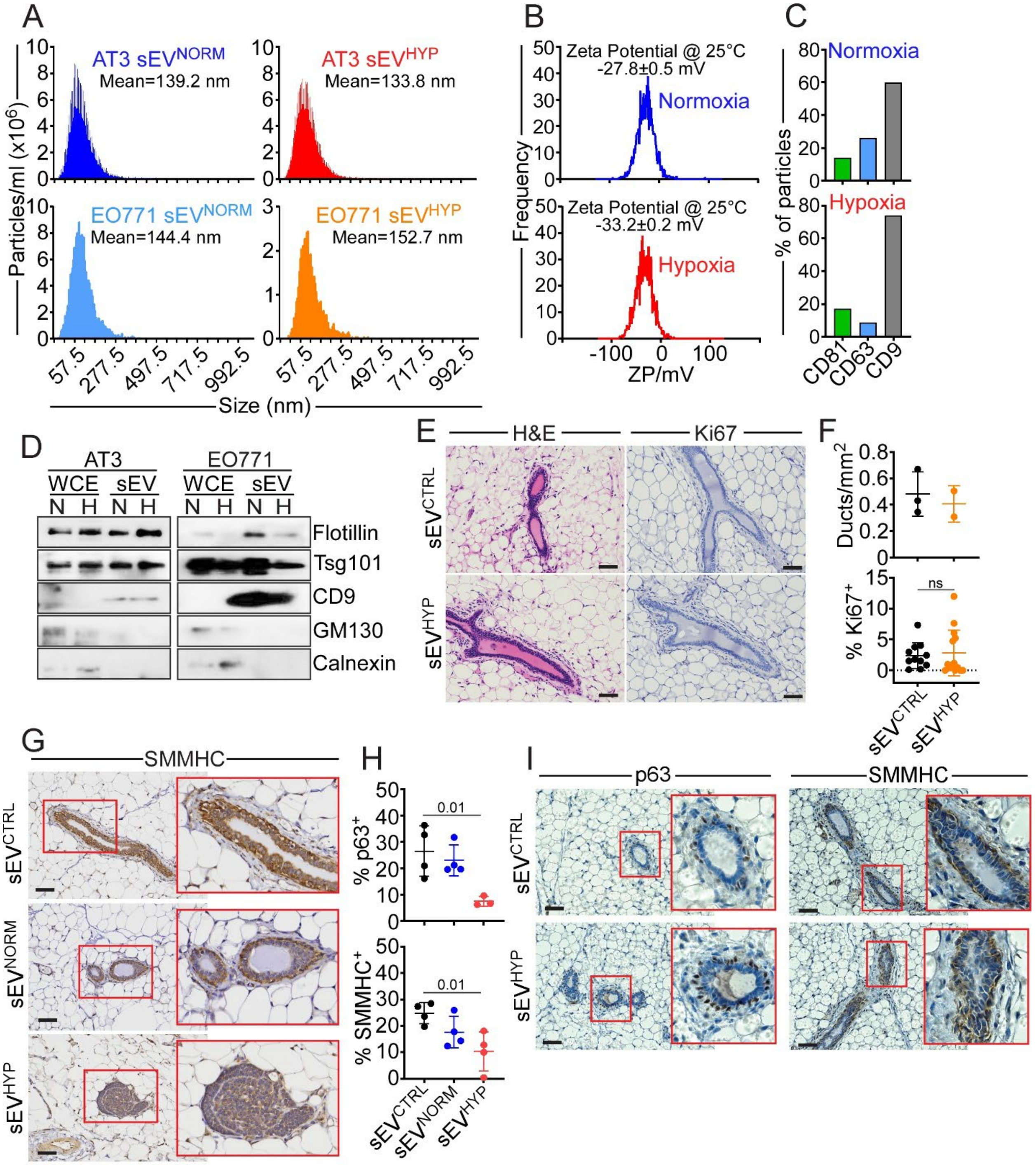

Supplemental Figure 1. sEV^{HYP}-induced mammary gland hyperplasia. (A) sEV isolated from AT3 or EO771 cells under normoxic (sEV^{NORM}) or hypoxic (sEV^{HYP}) conditions were characterized using a Zeta View analyzer with quantification of sEV number and size

distribution. Mean sEV values are indicated per each condition tested (n=6). (B) The conditions are as in (A) and zeta potential of AT3-derived sEV was quantified. *Blue*, normoxia; *red*, hypoxia. (n=3). (C) AT3 cell-derived sEV were analyzed for differential expression of sEV-associated tetraspanin molecules, CD81, CD63 and CD9 using an ExoView system (n=2). (D) Whole cell extracts (WCE) or sEV isolated from AT3 or EO771 cells were analyzed by Western blotting (n=3). (E) EO771 cell-derived sEV^{HYP} were injected in the abdominal mammary gland of immunocompetent C57BL/6 female mice and tissue samples harvested after 6 weeks were analyzed by H&E staining and Ki67 reactivity by immunohistochemistry (IHC). Representative images (n=3). Scale bar, 100 µm. (F) The conditions are as in (E) and the number of mammary gland ducts per area and percentage of $Ki67^+$ cells was quantified. Ctrl, control. Mean±SD. Numbers correspond to p values by one-way Anova with Tukey's multiple comparisons test. (G and H) AT3 cell-derived sEV were injected in the abdominal mammary gland of C57BL/6 mice and tissue samples harvested after 6 weeks were analyzed by IHC (G, representative images) and the percentage of $SMMHC^+$ and $p63^+$ cells was quantified (H). Scale bar, 100 µm. Mean±SD. Numbers correspond to p values by one-way Anova with Tukey's multiple comparisons test. (I) The conditions are as in (E) except that mammary gland tissues were injected with EO771 cell-derived sEV and analyzed after 6 weeks for expression of p63 and SMMHC, by IHC. Representative images. Scale bar, 100 µm.

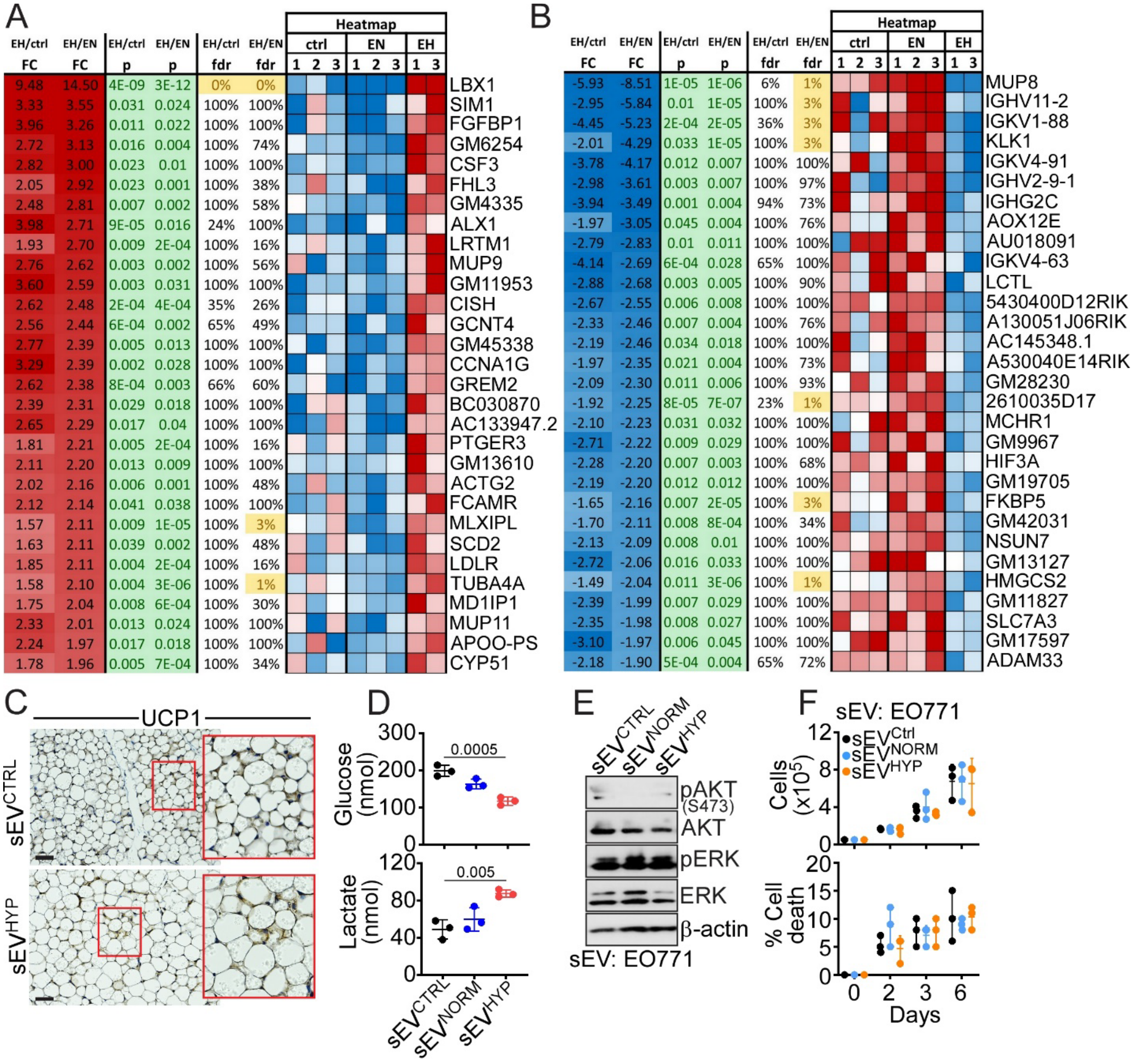

Supplemental Figure 2. sEV^{HYP}-induced metabolic reprogramming of the mammary gland. (A and B) AT3 cell-derived sEV were injected in the abdominal mammary gland of C57BL/6 female mice and analyzed for transcriptional changes by RNA-Seq. The top 30 genes upregulated (A) or downregulated (B) in sEV^{HYP} (EH)-treated samples compared to sEV^{CTRL} (ctrl) or sEV^{NORM} (EN) are shown in a heatmap. Fold change (FC) and p values are indicated. (C) EO771 cell-derived sEV^{HYP} were injected in the abdominal mammary gland of C57BL/6 mice and tissue samples harvested after 6 weeks were analyzed for UCP1 expression by IHC.

Representative images. Scale bar, 100 µm. Mean±SD (n=3). (D) Mouse primary mammary epithelial HC11 cells were incubated with AT3 cell-derived sEV and analyzed for glucose content (*top*) and lactate production (*bottom*). Mean±SD (n=3). Numbers correspond to p values by one-way Anova with Tukey's multiple comparisons test. (E) HC11 cells were incubated with EO771 cell-derived sEV and analyzed after 3 d by Western blotting. p, phosphorylated. (n=3). (F) The conditions are as in (E) and sEV-treated HC11 cells were analyzed for cell proliferation (*top*) and cell death (*bottom*) at the indicated time intervals by direct cell counting. Mean±SD (n=3).

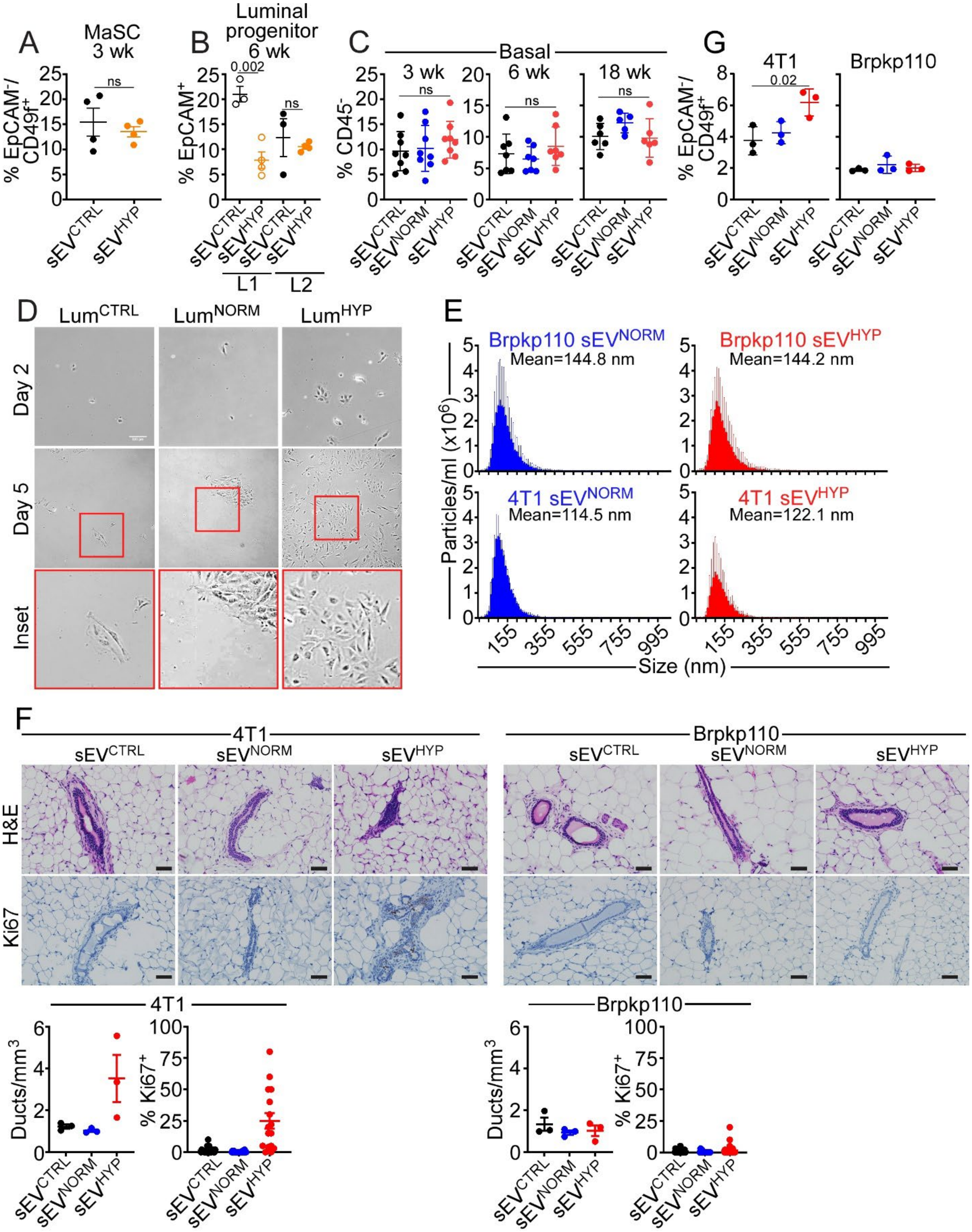

Supplemental Figure 3. sEV^{HYP}-regulation of mammary gland differentiation. (A and B)

EO771 cell-derived sEV^{HYP} were injected into the abdominal mammary gland of C57BL/6 mice

and the percentage of mammary stem cells (MaSC, A, n=4) and L1 or L2 luminal progenitor cells (B, n=3) were quantified after 3 and 6 weeks (wk), respectively, by flow cytometry. Mean±SD. Numbers correspond to p values by one-way Anova with Tukey's multiple comparisons test. (C) AT3 cell-derived sEV were injected into the abdominal mammary gland of C57BL/6 mice and the percentage of basal cells was quantified after 3, 6 and 18 weeks (wk), by flow cytometry. Mean±SD (n=8). Data were analyzed by one-way Anova with Tukey's multiple comparisons test. ns, not significant. (D) Luminal cells isolated from mouse mammary glands injected with AT3 cell-derived sEV were analyzed for cell proliferation. Representative images (n=3). Scale bar, 100 μm. Inset, magnification of indicated areas. (E) sEV isolated from metastatic 4T1 or non-metastatic Brpkp110 breast adenocarcinoma cells under normoxic (sEV^{NORM}, blue) or hypoxic (sEV^{HYP}, red) conditions were characterized using a Zeta View analyzer with quantification of sEV number and size distribution. Mean sEV values are indicated per each condition tested (n=3). (F) 4T1 or Brpkp110 cell-derived sEV were injected into the abdominal mammary gland of C57BL/6 mice and tissue samples harvested after 3 weeks were analyzed by H&E staining and immunohistochemistry with an antibody to Ki67 (*top*, representative images) with quantification of the number of mammary gland ducts and percentage of $Ki67^+$ cells (*bottom*). Mean±SEM (n=3). (G) The conditions are as in (F) and the percentage of mammary stem cells (MaSC) was quantified after 3 weeks (wk), by flow cytometry. Mean±SD (n=3). Numbers correspond to p values by one-way Anova with Tukey's multiple comparisons test.

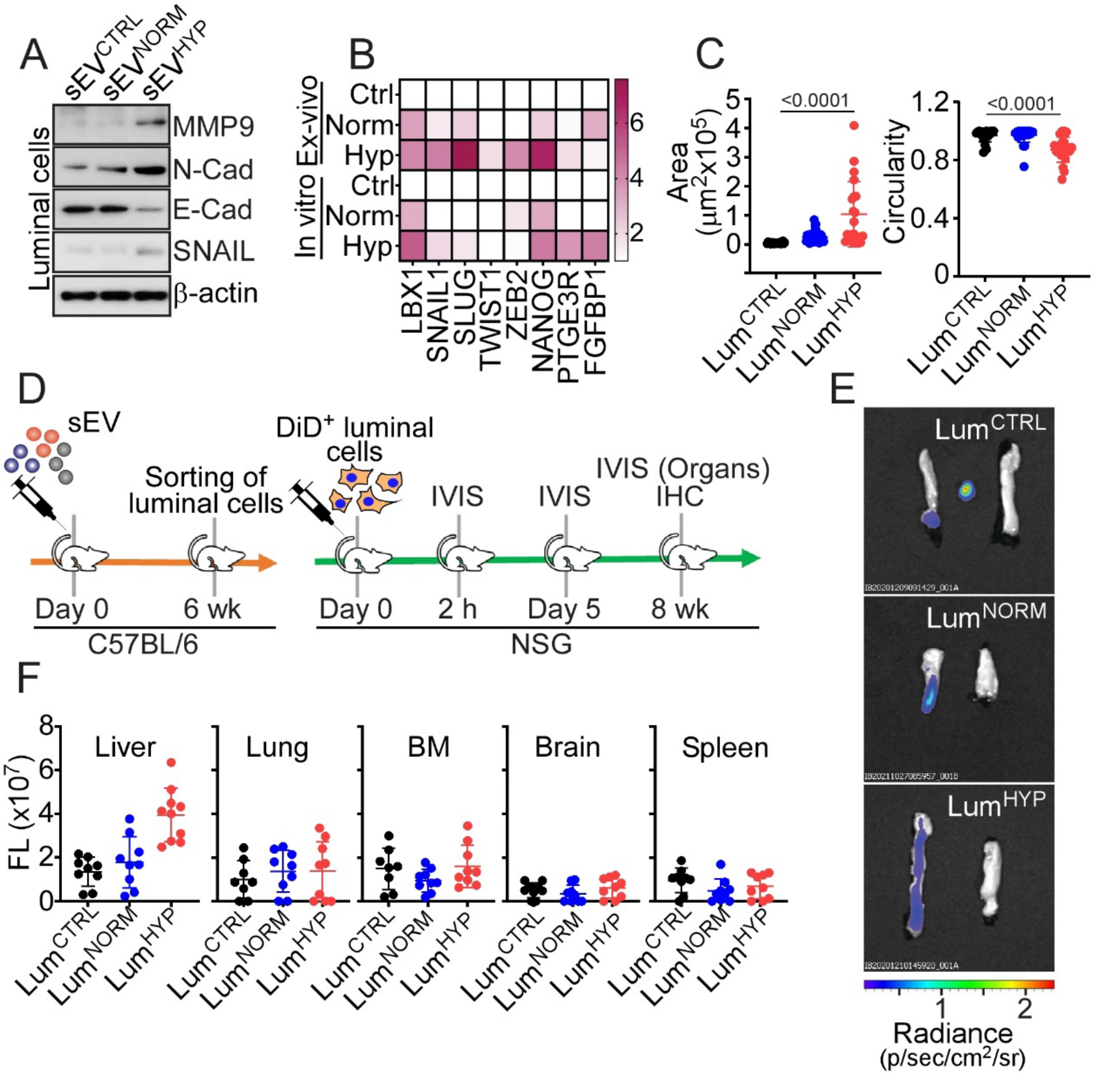

Supplemental Figure 4. **sEV^{HYP} regulation of mammary gland luminal cell compartment**. (A) Luminal cells isolated from mouse mammary glands injected with the various AT3 cell-derived sEV were analyzed by Western blotting (n=3). (B) Luminal cells as in (A) were analyzed for differential expression of EMT-related genes after sorting (ex-vivo) or after 1 week in culture (in vitro) by q-RT-PCR. Expression data are represented as a heatmap (n=3). (C) sEV-stimulated, sorted luminal cells as in (D) were analyzed for colony formation in Matrigel with quantification of surface area (*left panel*) and circularity (*right panel*). Mean±SD (n=3). Numbers correspond to p values by one-way Anova with Tukey's multiple comparisons test. (D) Schematic diagram of experimental timeline. Luminal cells isolated from the mammary gland of

C57BL/6 mice 6 weeks after sEV injection were stained with Vybrant-DiD dye, transferred to the abdominal mammary gland of immunocompromised NOD-SCID IL2Rγ recipient mice and organs harvested after 8 weeks were analyzed for DiD^+ cells using an IVIS® SpectrumCT In Vivo Imaging System. (E) The conditions are as in (D) and mammary glands were analyzed for the presence of DiD^+ luminal cells using an IVIS® SpectrumCT In Vivo Imaging System (representative 3D-reconstructed images). (F) The conditions are as in (E) and the indicated tissue samples were analyzed 8 weeks after sEV injection for the presence of DiD^+ luminal cells using an IVIS® SpectrumCT In Vivo Imaging System and quantfied. Mean±SD (n=9).

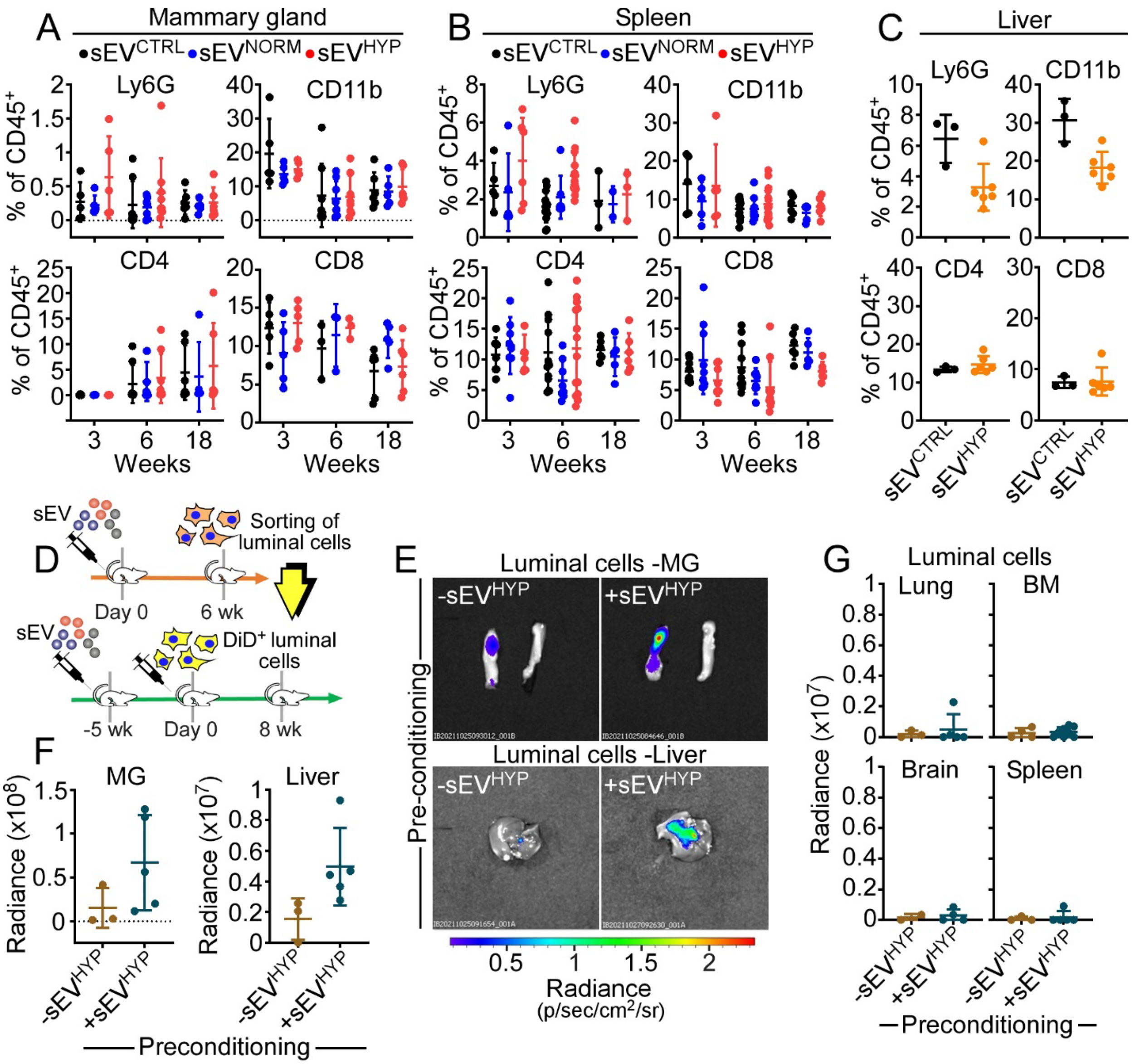

Supplemental Figure 5. **sEV^{HYP}** mediated immune reprogramming, **in vivo**. (A and B) Mammary gland tissues (A) or spleen samples (B) collected from C57BL/6 mice injected with the indicated AT3 cell-derived sEV were analyzed for myeloid (LY6G^{+}, CD11b^{+}) or lymphoid (CD4^{+}, CD8^{+}) immune cell populations at the indicated time intervals, by flow cytometry. Mean±SD. (C) C57BL/6 mice were injected with the indicated EO771 cell-derived sEV and liver samples were analyzed after 6 weeks for changes in myeloid and lymphoid cell populations, by flow cytometry. Mean±SD (n=3). (D) Schematic diagram of reconstitution experiments by pre-

conditioning of C57BL/6 immunocompetent mice with the various sEV 5 weeks before mammary gland injection of isolated luminal cells. (E) The conditions are as in (D) and mammary gland (*top*) or liver (*bottom*) samples harvested from reconstituted C57BL/6 mice without or with sEV^{HYP} preconditioning were analyzed for the presence of DiD^{+} luminal cells using an IVIS® SpectrumCT In Vivo Imaging System. Representative 3D-reconstructed images. (F) The conditions are as in (E) and luminal cell-associated fluorescence imaging was quantified in the mammary gland (MG) or liver samples per each condition tested 8 weeks after reconstitution. Mean±SD (n=5). (G) The conditions are as in (D) and luminal cell-associated fluorescence imaging was quantified in the indicated organs 8 weeks after reconstitution. Mean±SD (n=5).

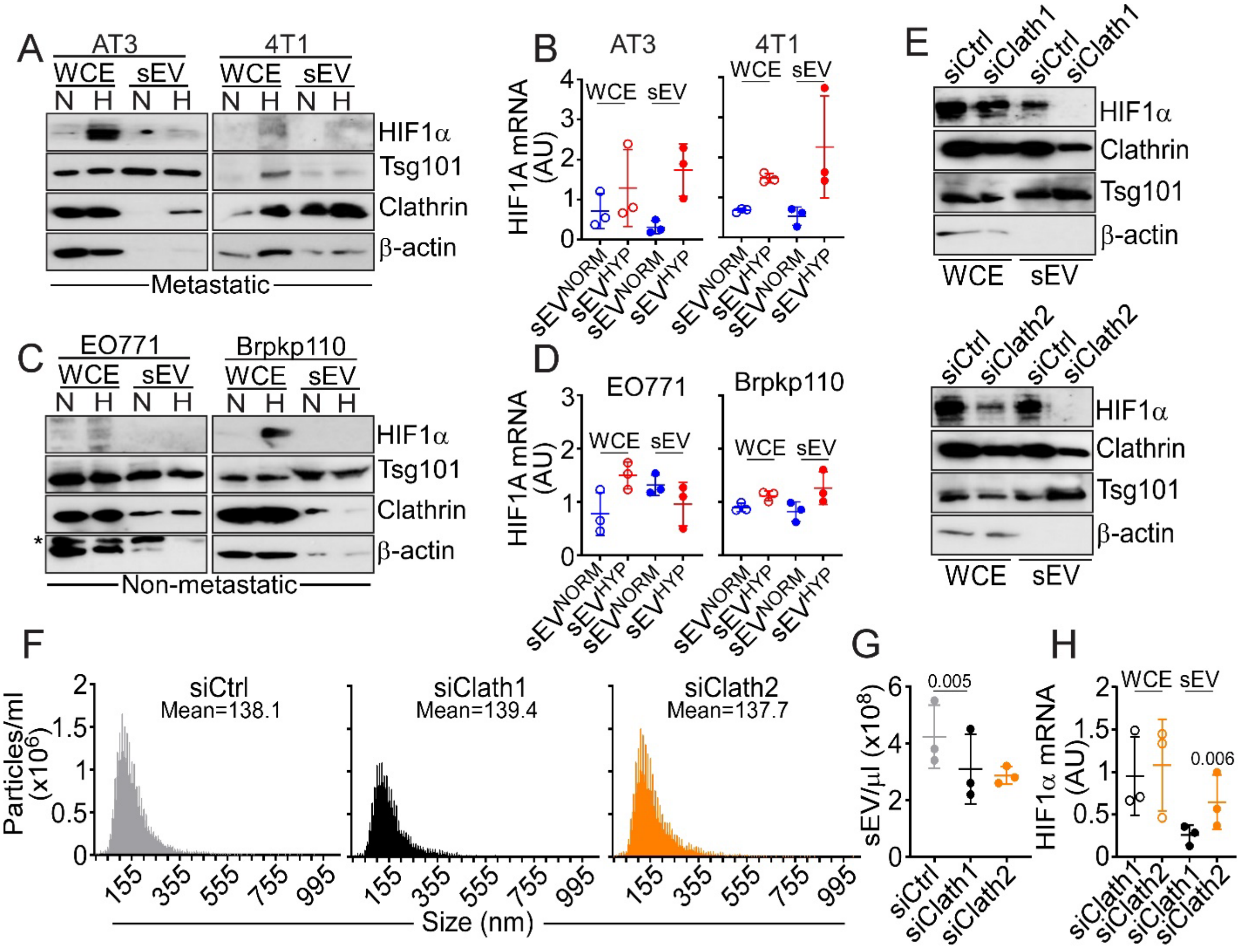

Supplemental Figure 6. **Clathrin-dependent loading of HIF1a in sEV^{HYP}**. (A and B) Metastatic breast adenocarcinoma AT3 or 4T1 cells were analyzed by Western blotting (A) or differential expression of HIF1a mRNA by qRT-PCR (B). WCE, whole cell extracts. Mean±SD (n=3). (C and D) The conditions are as in (A and B) except that non-metastatic breast adenocarcinoma EO771 or Brpkp110 cells were analyzed by Western blotting (C) or qRT-PCR (D). (E) AT3 cells were transfected with two independent clathrin-directed siRNA (siClath1, *top*; siClath2, *bottom*) and analyzed by Western blotting. (F and G) sEV isolated from AT3 cells transfected with control siRNA or two independent clathrin-directed siRNA were characterized using a Zeta View analyzer with quantification of sEV size distribution (F) and yield (G). Mean

sEV values are indicated per each condition tested. (H) The conditions are as in (E) and siRNA-transfected AT3 cells were analyzed for changes in HIF1a mRNA expression in WCE or sEV by qRT-PCR. Mean±SD (n=3). Numbers correspond to p values by one-way Anova with Tukey's multiple comparisons test.

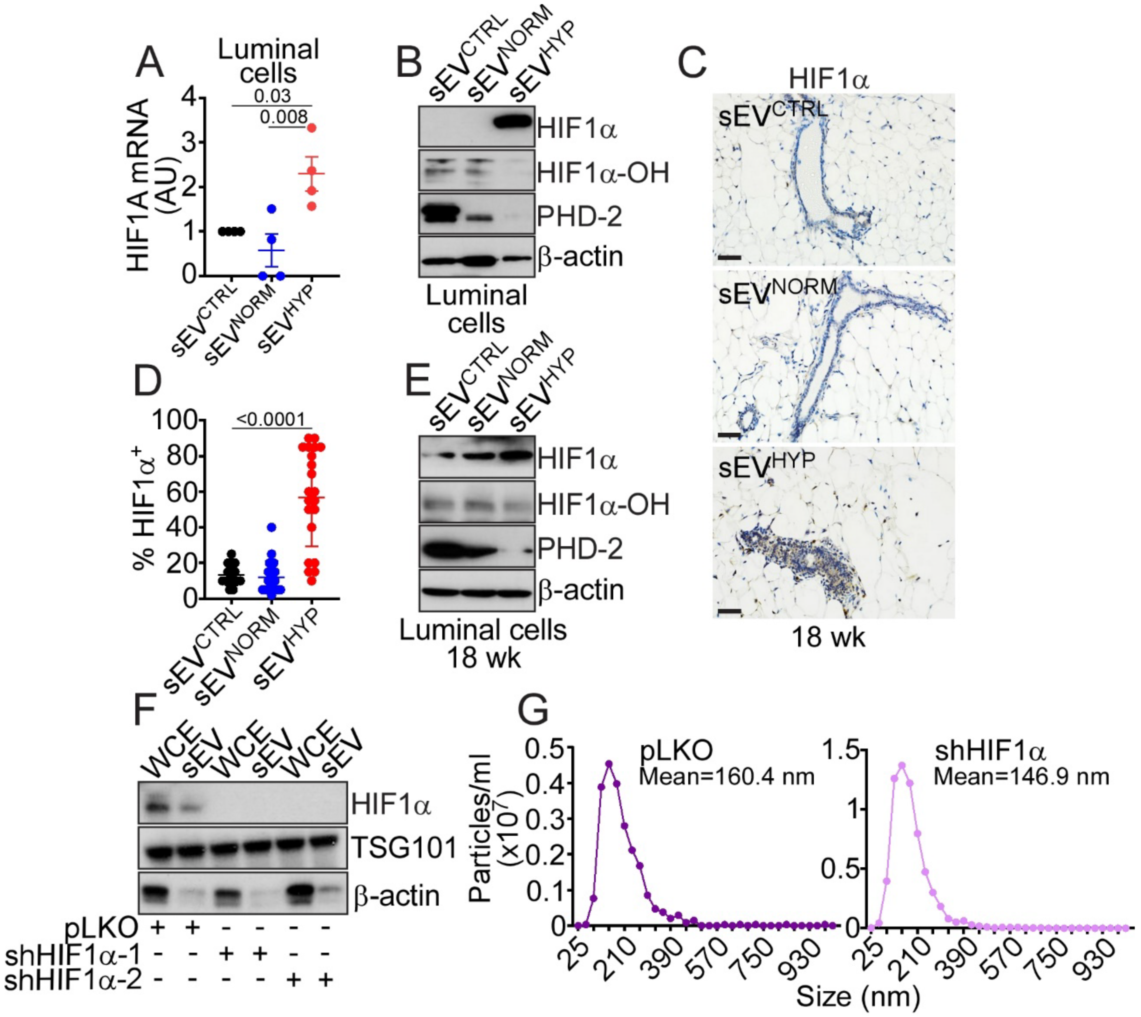

Supplemental Figure 7. **sEV^{HYP}-HIF1α stimulation of mammary gland angiogenesis**. (A) Luminal cells sorted from mammary glands 6 weeks after injection with the various AT3 cell-derived sEV were analyzed for HIF1α mRNA expression by q-RT-PCR. Mean±SD (n=4). Numbers correspond to p values by one-way Anova with Tukey's multiple comparisons test. (B) The conditions are as in (A) and protein extracts were analyzed by Western blotting. (C and D) C57BL/6 mice were injected in the abdominal mammary gland with AT3 cell-derived sEV and analyzed for HIF1α expression by IHC after 18 weeks (C) and quantified (D). Mean±SD (n=4). Numbers correspond to p values by one-way Anova with Tukey's multiple comparisons test. (E) The conditions are as in (C and D) and luminal cells sorted after 18 weeks from sEV injection

were analyzed by Western blotting. (F) Whole cell extracts (WCE) or sEV from stable clones of AT3 cells transduced with pLKO or two independent HIF1a-directed shRNA were analyzed by Western blotting (n=3). (G) sEV^{HYP} isolated from AT3 cells transduced with pLKO or shHIF1α were analyzed on a Zeta View analyzer with quantification of sEV number and size distribution. Mean sEV values are indicated per each condition tested.

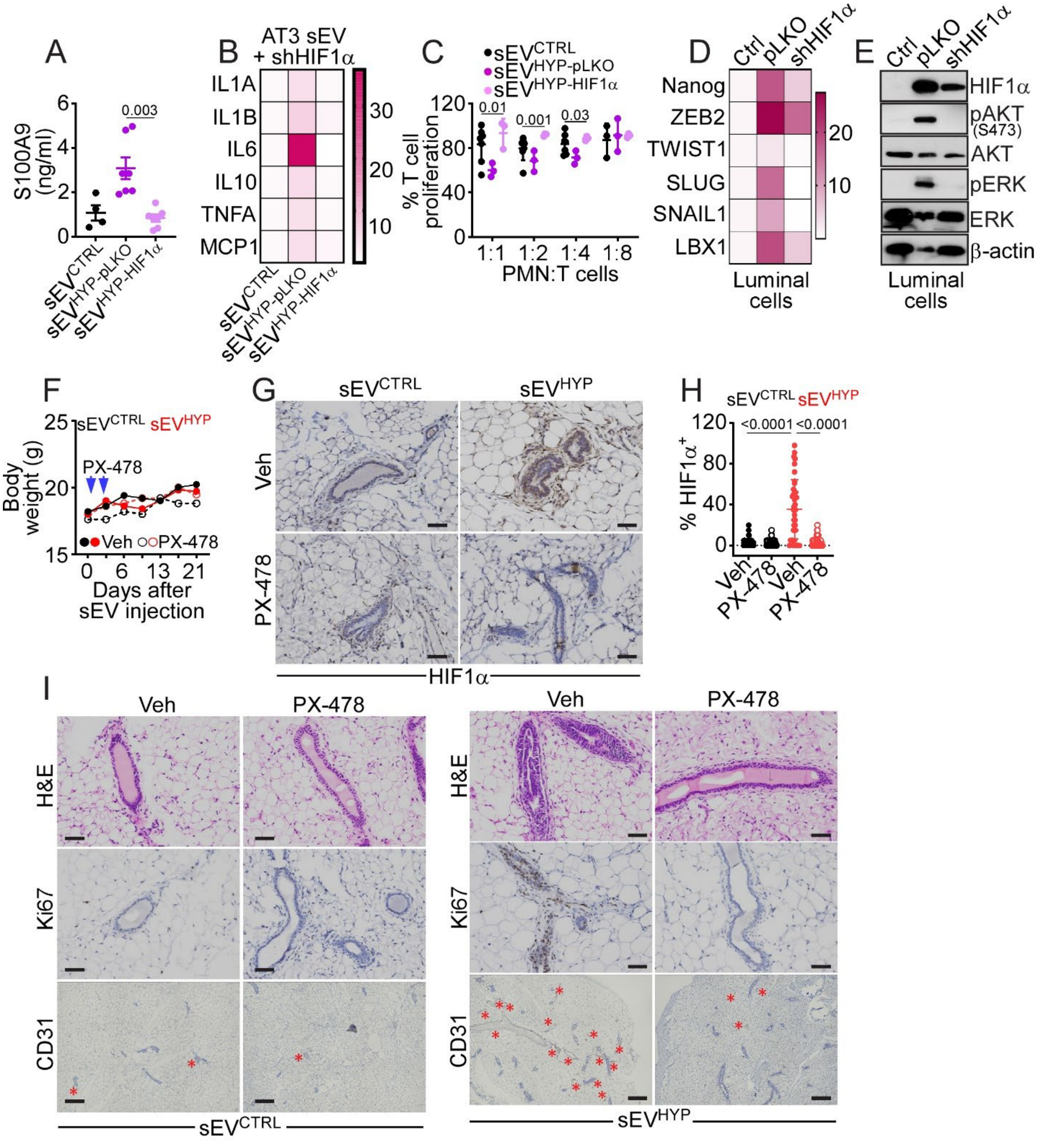

Supplemental Figure 8. **sEV^{HYP}-HIF1α modulation of T cell responses and mammary gland development**. (A) Plasma samples from C57BL/6 mice injected with AT3 cell-derived sEV^{HYP} without (sEV$^{HYP\text{-}pLKO}$) or with stable HIF1a silencing (sEV$^{HYP\text{-}HIF1\alpha}$) were analyzed for circulating S100A9 levels, by ELISA. Mean±SD (n=7). Numbers correspond to p values by one-

way Anova with Tukey's multiple comparisons test. (B) Aliquots of PMN isolated from C57BL/6 mice were incubated with AT3 cell-derived sEV^{HYP} without ($sEV^{HYP\text{-}pLKO}$) or with stable HIF1a silencing ($sEV^{HYP\text{-}HIF1\alpha}$) and analyzed for changes in the expression of the indicated cytokines by qRT-PCR. Data are expressed as a heatmap of a representative experiment (n=3). (C) PMN isolated from C57BL/6 mice and treated as in (B) were mixed with syngeneic T cells at the indicated ratios and T cell proliferation was quantified. Numbers correspond to p values by one-way Anova with Tukey's multiple comparisons test (n=3). (D) Mammary gland luminal cells sorted from C57BL/6 mice injected with AT3 cell-derived sEV^{HYP} as in (A) were analyzed for changes in expression of EMT-associated genes, by q-RT-PCR. Data are expressed as a heatmap of a representative experiment (n=3). (E) The conditions are as in (D) and sorted luminal cells were analyzed by Western blotting. p, phosphorylated. (F) Mouse body weight quantified at the indicated time intervals after administration (arrows) of vehicle (closed symbols) or PX-478 (open symbols) on d. 1 and d.3. (G and H) Mice treated with vehicle (Veh) or PX-478 were analyzed after 3 weeks for HIF1α expression by IHC (G, representative images) and quantified (H). Scale bar, 100 μm. Mean±SD. Numbers correspond to p values by one-way Anova with Tukey's multiple comparisons test. (I) Animals treated with Veh or PX-478 as in (F) were harvested after 3 weeks and analyzed by H&E staining and expression of Ki67 or CD31 by IHC. Representative images (n=3). Scale bar, 100 μm.

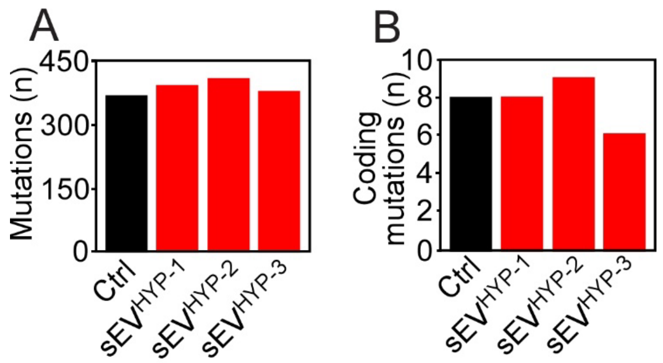

Supplemental Figure 9. Mammary gland whole genome sequencing after sEV^{HYP} injection. (A and B) The total number of mutations (A) and protein-changing mutations (B) was determined by whole genome sequencing of control or three independent samples of isolated luminal cells exposed to sEV^{HYP}, in vivo.
